# Nitrosative stress induced by homocysteine thiolactone drives vascular cognitive impairments via GTP cyclohydrolase 1 S-nitrosylation *in vivo*

**DOI:** 10.1016/j.redox.2022.102540

**Published:** 2022-11-13

**Authors:** Ya-Ling Yin, Yuan Chen, Feng Ren, Lu Wang, Mo-Li Zhu, Jun-Xiu Lu, Qian-Qian Wang, Cheng-Biao Lu, Chao Liu, Yong-Ping Bai, Shuang-Xi Wang, Jian-Zhi Wang, Peng Li

**Affiliations:** a Sino-UK Joint Laboratory of Brain Function and Injury and Department of Physiology and Neurobiology, School of Basic Medical Sciences, Xinxiang Medical University, Xinxiang, China; b The Key Laboratory of Cardiovascular Remodeling and Function Research, Chinese Ministry of Education, Chinese National Health Commission and Chinese Academy of Medical Sciences, The State and Shandong Province Joint Key Laboratory of Translational Cardiovascular Medicine, Qilu Hospital of Shandong University, Jinan, Shandong, China; c School of Pharmacy, Henan International Joint Laboratory of Cardiovascular Remodeling and Drug Intervention, Xinxiang Medical University, Xinxiang, Henan, China; d Hubei Key Laboratory of Cardiovascular, Cerebrovascular, and Metabolic Disorders, Hubei University of Science and Technology, Xianning, China; e Department of Geriatric Medicine and Department of Cardiovascular Medicine, Coronary Circulation Center, National Clinical Research Center for Geriatric Disorders, Xiangya Hospital, Central South University, Changsha, Hunan, China; f Department of Pathophysiology, School of Basic Medicine and the Collaborative Innovation Center for Brain Science, Key Laboratory of the Ministry of Education of China for Neurological Disorders, Tongji Medical College, Huazhong University of Science and Technology, Wuhan, Hubei, China

**Keywords:** GTP cyclohydrolase 1, S-nitrosylation, Endothelial cell, Vascular cognitive impairment, Nitrosative stress

## Abstract

**Background:**

s: Hyperhomocysteinemia (HHcy) is one of risk factors for vascular cognitive impairment (VCI). GTP cyclohydrolase 1 (GCH1) deficiency is critical to oxidative stress in vascular dysfunction. The aim of this study was designed to examine whether HHcy induces VCI through GCH1 S-nitrosylation, a redox-related post-translational modification of cysteine.

**Methods:**

The VCI model was induced by feeding mice homocysteine thiolactone (HTL) for 16 consecutive weeks. The cognitive functions were evaluated by step-down avoidance test, passive avoidance step-through task test, and Morris water maze (MWM) test. Protein S-nitrosylation was assayed using a biotin-switch method.

**Results:**

In cell-free system, nitric oxide (NO) donor induced GCH1 protein S-nitrosylation and decreased GCH1 activity. In endothelial cells, HTL increased GCH1 S-nitrosylation, reduced tetrahydrobiopterin, and induced oxidative stress, which were attenuated by N-acetyl-cysteine, L-N6-1-Iminoethyl-lysine, mutant of GCH1 cysteine 141 to alanine (MT-GCH1) or gene deletion of inducible NO synthase (iNOS). Further, HTL incubation or iNOS overexpression promoted endothelial cellular senescence, but abolished by exogenous expression of MT-GCH1 or pharmacological approaches including N-acetyl-cysteine, L-sepiapterin, and tempol. In wildtype mice, long-term administration of HTL induced GCH1 S-nitrosylation and vascular stiffness, decreased cerebral blood flow, and damaged the cognitive functions. However, these abnormalities induced by HTL administration were rescued by enforced expression of MT-GCH1 or gene knockout of iNOS. In human subjects, GCH1 S-nitrosylation was increased and cognitive functions were impaired in patients with HHcy.

**Conclusion:**

The iNOS-mediated nitrosative stress induced by HTL drives GCH1 S-nitrosylation to induce cerebral vascular stiffness and cognitive impairments.

## 1 Introduction

Vascular cognitive impairment (VCI), due to hypoperfusion, accounts for at least 20% of cases of dementia caused by cerebrovascular diseases, secondary to Alzheimer's disease [1]. Hyperhomocysteinemia (HHcy), as a well-known independent risk factor of atherosclerosis, also increases the risk of VCI and vascular dementia [2]. Serum homocysteine level is inversely related to cognitive functions in patients with dementia, and the elevation is more common among VCI patients than Alzheimer's disease patients [3]. Although a linkage between HHcy and cognitive impairments has been established, the underlying molecular mechanism is elusive.

Dysfunction of cerebrovascular endothelial cells is usually the foremost in bearing the attack of hypoperfusion, which is a prelude to VCI and vascular dementia [4]. Endothelial senescence is the main determinant of endothelial dysfunction and thus of age-related cardiovascular diseases [5,6]. Nitric oxide (NO) produced by endothelial NO synthase (eNOS) is essential for cardiovascular homeostasis owing to its anti-inflammatory, anti-thrombotic, anti-proliferative, and antioxidant effects [7]. The eNOS must be fully saturated with tetrahydrobiopterin (BH4) to completely couple NADPH oxidation to NO production [8]. Otherwise, eNOS functions in an “uncoupled” state in which NAD(P)H-derived electrons are added to molecular oxygen, leading to the production of reactive oxygen species (ROS). As the rate-limiting enzyme of de novo BH4 synthesis, the critical role of GTP cyclohydrolase 1 (GCH1) in maintaining eNOS function and preventing endothelial dysfunction has been established by us. For example, downregulation of GCH1 by small RNA inference mediated acute gene silence, hyperglycemia, HHcy or dyslipidemia causes BH4 deficiency and uncouples eNOS *in vitro* and *in vivo* [[9], [10], [11]]. However, the role of GCH1 in HHcy-induced VCI remains unknown.

As a posttranslational modification of protein, S-nitrosylation is a ubiquitous redox-related modification of cysteine thiol under nitrosative stress [12,13]. It is now regarded as a selective and specific signal controlled by intracellular NO, and has been close associated with protein localization, stability, and function [14,15]. As reported, protein S-nitrosylation plays critical roles in cognitive declines in Alzheimer's disease, diabetes and cardiovascular diseases [[16], [17], [18]]. In light of these observations, we speculate that HHcy may induce cerebrovascular hypoperfusion and cognitive impairments through GCH1 S-nitrosylation. Here, we reported that HHcy or homocysteine thiolactone (HTL), a major metabolite of homocysteine [10], triggered nitrosative stress to induce GCH1 S-nitrosylation at cysteine 141 via inducible NO synthase (iNOS). In this way, HTL-induced GCH1 inhibition promotes endothelial cell senescence and VCI. In perspective, pharmacological approaches, e.g. S-nitrosylation blockage, iNOS inhibition, BH4 supplementation, and ROS clearance, may prevent VCI in HHcy patients.

## 2 Materials and Methods

An expanded section of Materials and Methods is available in the Online Supplement.

### 2.1 Establishment of HHcy model by HTL administration

The VCI model was mimicked by intragastrically feeding mice with HTL (100 mg/kg/day, 1 ml/kg per two days) for 16 consecutive weeks [10].

### 2.2 Step-down avoidance test

Mice were trained in a step-down avoidance test on the tenth day after each stress exposure. Mice were placed on a 7 × 25 × 2.5 cm platform. The platform faced a 42 × 25 cm grid of parallel stainless steel bars that were 0.1 cm in caliber and spaced 1 cm apart. In the training sessions, the animals received a 0.5 mA scramble foot shock for 2 s immediately upon stepping down. The interval of time that elapsed until the mice stepping down and placing all four paws on the grid was defined as the latency. Mice were excluded from the experiment if the waiting time is more than 300 s on the platform during the training in step-down avoidance test. Because we used a single trail based on the animal response, but not multiple trails with a 10-s interval [[19], [20], [21]], it is appropriate to measure long-term memory at the 24th hour after training. The single trail was performed under the conditions of electric current = 0.1–0.5 mA, frequency = 1 Hz, and time of duration = 2–15 s. Then, the mouse was again placed on a platform the latency and number of errors the mice stepped down the platform and were shocked were recorded.

### 2.3 Passive avoidance step-through task test

This procedure was carried out as previously described, with some modifications [22]. The apparatus consisted of a light compartment illuminated with a lamp (60 W positioned above the apparatus) and a dark compartment (20 × 20 × 40 cm) with an electrifiable grid floor. The two compartments were separated by a black partition with a rectangular doorway (8 × 8 cm). The floor was constructed of stainless-steel grids 0.2 cm in diameter and at 0.8 cm intervals. Intermittent electric shock (50 Hz, 10 s, 0.5 mA intensity) was delivered to the grid floor of the dark compartment by an isolated stimulator.

An acquisition trial was performed for mice to habituate in both compartments freely for 3 min. During training, each mouse was placed in light compartment, Once the mouse crossed with all four paws into the dark compartment, the door was closed and foot shock was administered. Three minutes later, the mouse was removed from the apparatus and returned to its cage. The mouse waited for more than 100 s to cross to the dark compartment were excluded from the experiment. Twenty-four hours after training, the mouse was again placed in light compartment, the latency to enter the dark compartment with all four paws and number of errors cross into the dark compartment were timed. In the test phase, the latency of mice that did not cross the door was identified as 300 s.

### 2.4 Morris water maze (MWM) test

Spatial learning and memory were tested by MWM as we described previously [[23], [24], [25]]. The widely used protocols of MWM test are 1 training per day lasting 6 consecutive days and 2 trainings per day lasting 3 consecutive days. Others liking 1 training per day lasting 4 consecutive days and 1 training per day lasting 5 consecutive days are also used. The threshold of the training is that mice are able to find the platform correctly. We chose the protocol of 1 training per day lasting 6 consecutive days. During the training phase, one week before the end of the experiments, spatial learning and memory were assessed in an MWM (150 cm in diameter, 50 cm-high) filled with white water (22 °C) and surrounded with distal extramaze cues. Before being trained, animals were handled for 1 min a day for 2 days. Mice were then familiarized with water and swimming during two familiarization days (day 1 and day 2) where they had to find a visible platform in the center of a small pool (60 cm diameter) surrounded with curtains (three consecutive trials a day; 60 s-cut-off). On day 0, to evaluate visuomotor deficits, mice were given six trials (90 s-cut-off) to find a visible platform pointed out with a cue in the Morris water maze that was surrounded with white curtains. During the training sessions (days 1–4), animals were required to locate the submerged platform by using distal extramaze cues. They were trained for six trials a day (90 s-cut-off) with an intertrial interval of 5 min for four consecutive days. In order to facilitate spatial learning, mice were introduced from four different starting points, in a randomized daily order. The swimming path and the time used to find the platform were recorded by a video camera fixed on the ceiling of the room, 1.5 m from the water surface. During the probe test, the platform was removed from the pool and spatial memory was evaluated for 60 s. The latency that is the time spent by mice first reaching the location of platform, the percentage of time spent in the target quadrant and the number of platform crossings were recorded.

### 2.5 Protein S-nitrosylation assay

Protein was extracted according to the manufacturer's specification S-nitrosylated Protein Detection Assay Kit (Cayman, USA), which is based on the “Biotin-switch” method as described previously [12].

### 2.6 Measurements of ROS *in vitro* and *in vivo*

As recommended by Michael P. Murphy et al. [26], we measured intracellular superoxide using DHE, hydrogen peroxide by Amplex Red, mitochondrial peroxynitrite by MitoPY1, and *in vivo* lipid oxidation by F2-isoprostanes.

## 3 Statistical analysis

Data are reported as mean ± SEM. Tukey's HSD test is used if the sample numbers are identical. Scheffe test is used if the sample numbers are not identical. Dunnett test is used if all other groups compared with control group only. Bonferroni correction is used to reduce type 1 error. P < 0.05 was considered as significant.

## 4 Results

### 4.1 NO donor induces recombinant GCH1 protein S-nitrosylation *in vitro*

To investigate if GCH1 protein is S-nitrosylated by NO, we firstly performed analyses of amino acid sequence to identify the potential sites because S-nitrosylation is an NO-directed modification of cysteine thiol [27]. As seen in Online Figs. S1A and S1B, GCH1 proteins are highly conserved between human and mouse. In human GCH1 protein, the 18th, 141th, and 212th amino acids are cysteine (C18/C141/C212), equal to C9/C132/C203 in mouse GCH1 protein, demonstrating GCH1 protein is able to be S-nitrosylated by NO.

**Fig. 1:**
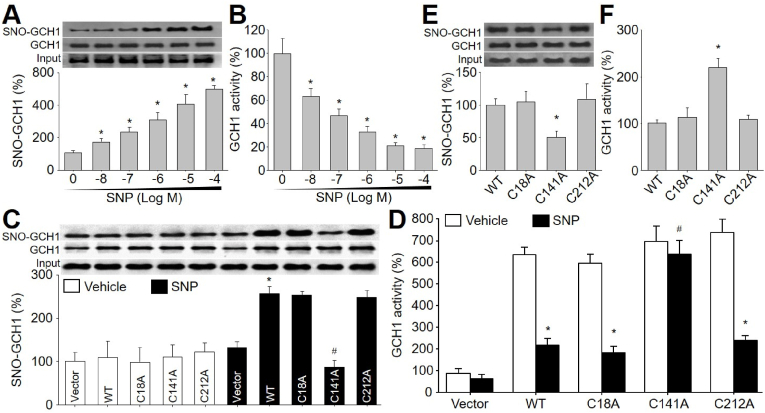
**GCH1 at cysteine 141 is S-nitrosylated by sodium nitroprusside (SNP), which inhibits GCH1 activity.** (**A** and **B**) Recombinant human GCH1 protein was incubated with SNP (0.001–10 μM) for 2 h in reaction buffers. Reaction products were subjected to determine GCH1 S-nitrosylation using biotin-switch method in **A** and GCH1 activity in **B**. N = 5 per group. *P < 0.05 *vs.* Vehicle (point 0). (**C** and **D**) HEK293 cells were transfected with plasmids expressing GCH1 (*WT*, C18A, C141A, C212A) for 48 h and then treated with SNP (1 μM) for 2 h. His-tagged GCH1 protein purified from total cell lysates was subjected to measure GCH1 S-nitrosylation in **C** and GCH1 activity in **D**. N = 5 per group. *P < 0.05 *vs.* Vehicle plus WT, C18A, or C212A. ^#^*P* < 0.05 *vs.* SNP plus *WT*. (**E** and **F**) Human umbilical vein endothelial cells were infected with adenovirus expressing GCH1 (*WT*, C18A, C141A, C212A) for 48 h and then treated with SNP (1 μM) for 2 h. Purified GCH1 protein from total cell lysates was subjected to measure GCH1 S-nitrosylation in **E** and GCH1 activity in **F**. N = 5 per group. *P < 0.05 *vs.* WT. A repeated-measures ANOVA following by Bonferroni correction was used to determine *P* value between two groups in **A** and **B**. A one-way ANOVA followed by Tukey's HSD test was used to determine *P* value between two groups in **C** and **D**. A one-way ANOVA followed by Dunnett test was used to determine *P* value between two groups in **E** and **F**.

We incubated recombinant human GCH1 protein with sodium nitroprusside (SNP), which is an NO donor [12]. As illustrated, the S-nitrosylated level of GCH1 protein was remarkably increased by SNP in a concentration-dependent manner, compared to vehicle-treated GCH1 protein (Fig. 1A). In contrary, GCH1 activity was decreased by SNP (Fig. 1B), showing that NO-directed S-nitrosylation suppresses GCH1 activity.

### 4.2 SNP inhibits GCH1 activity by modification of cysteine 141

To elucidate which cysteine is S-nitrosylated by NO, we generated plasmids expressing wildtype (*WT*) GCH1 (WT-GCH1) and mutated GCH1 (MT-GCH1-C18A, MT-GCH1-C141A, and MT-GCH1-C212A) with replacements of cysteine to alanine (C to A), and transfected these DNA constructs into HEK293 cells followed by SNP treatment. As demonstrated in Fig. 1C, SNP increased S-nitrosylated levels of WT-GCH1, MT-GCH1-C18A, and MT-GCH1-C212A, but not MT-GCH1-C141A. Accordingly, GCH1 activity in HEK293 cells expressing WT-GCH1, MT-GCH1-C18A or MT-GCH1-C212A were inhibited by SNP. While, MT-GCH1-C141A was resistant to SNP-induced reduction of GCH1 activity (Fig. 1D). As indicated in Fig. 1E and F, all results observed in KEK293 cells were replicated in human umbilical vein endothelial cells (HUVECs). In sum, these data indicate that NO inhibits GCH1 activity through S-nitrosylation at cysteine 141.

### 4.3 HTL induces GCH1 S-nitrosylation in endothelial cells via iNOS-mediated nitrosative stress

**Fig. 2:**
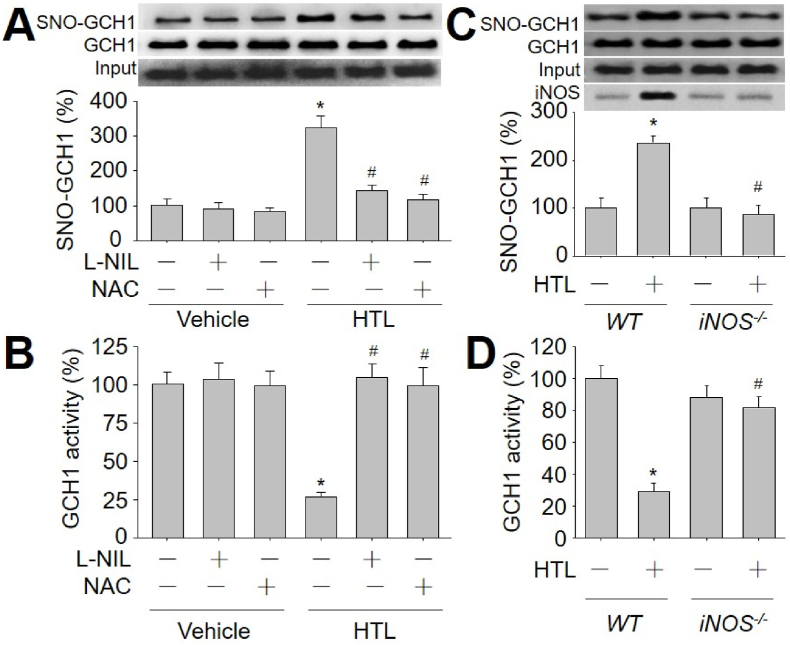
**Homocysteine thiolactone (HTL) induces GCH1 S-nitrosylation via iNOS signaling in endothelial cells.** (**A** and **B**) Human umbilical vein endothelial cells were pretreated with N-acetyl-cysteine (NAC, 2.5 mM) or L-N6-1-Iminoethyl-lysine (L-NIL, 1 mM) for 30 min followed by incubation with HTL (1 mM) for 24 h. Purified GCH1 protein from cell lysates was subjected to measure the S-nitrosylated level of GCH1 protein using biotin-switch method in **A** and GCH1 activity in **B**. N = 5 per group. *P < 0.05 *vs.* Vehicle alone. ^#^*P* < 0.05 *vs.* HTL alone. (**C** and **D**) Primary aortic endothelial cells isolated from wildtype (*WT*) mice and iNOS gene knockout (*iNOS*^*−/−*^) mice were incubated with HTL (1 mM) for 24 h. Total cell lysates were subjected to measure GCH1 S-nitrosylation in **C** and GCH1 activity in **D**. N = 5 per group. *P < 0.05 *vs. WT* cells. ^#^*P* < 0.05 *vs.* HTL-treated *WT* cells. A one-way ANOVA followed by Tukey's HSD test was used to determine *P* value between two groups in this figure.

We next examined if HTL induces GCH1 S-nitrosylation in HUVECs. As indicated in Fig. 2A and B, HTL increased GCH1 S-nitrosylation, and reduced GCH1 activity, compared to vehicle, suggesting that HHcy induces GCH1 S-nitrosylation to inhibit GCH1 activity in endothelial cells.

As reported, aberrant NO derived from iNOS contributes to multiple cerebrovascular diseases [28]. To examine whether HHcy induces GCH1 S-nitrosylation through iNOS-driven nitrosative stress, we used N-acetyl-cysteine (NAC) to block protein S-nitrosylation *in vivo* (Online Figs. S1C and S1D), which is a clinical drug used for chronic obstructive lung disease [29], and L-N6-1-Iminoethyl-lysine (L-NIL, Online Fig. S1C) to selectively inhibit iNOS. Both NAC at 2.5 mM and L-NIL at 1 mM, as reported previously reports [30,31], abolished GCH1 S-nitrosylation and reversed GCH1 activity in HTL-treated cells (Fig. 2A and B), demonstrating that HTL inhibits GCH1 activity through iNOS-mediated NO-directed S-nitrosylation.

To exclude any potential off-target effects of L-NIL, we determined if genetic deletion of iNOS (*iNOS*^*−/−*^) mimicked the effects of L-NIL on GCH1 S-nitrosylation. Primary murine aortic endothelial cells isolated from *WT* mice and *iNOS*^*−/−*^ mice were incubated with HTL. Similarly, HTL increased GCH1 S-nitrosylation and decreased GCH1 activity in *WT* cells, but not in *iNOS*^*−/−*^ cells (Fig. 2C and D), further supporting the concept that HTL via iNOS increases GCH1 S-nitrosylation in endothelial cells.

### 4.4 HTL-induced GCH1 inhibition and oxidative stress are S-nitrosylation dependent in HUVECs

**Fig. 3:**
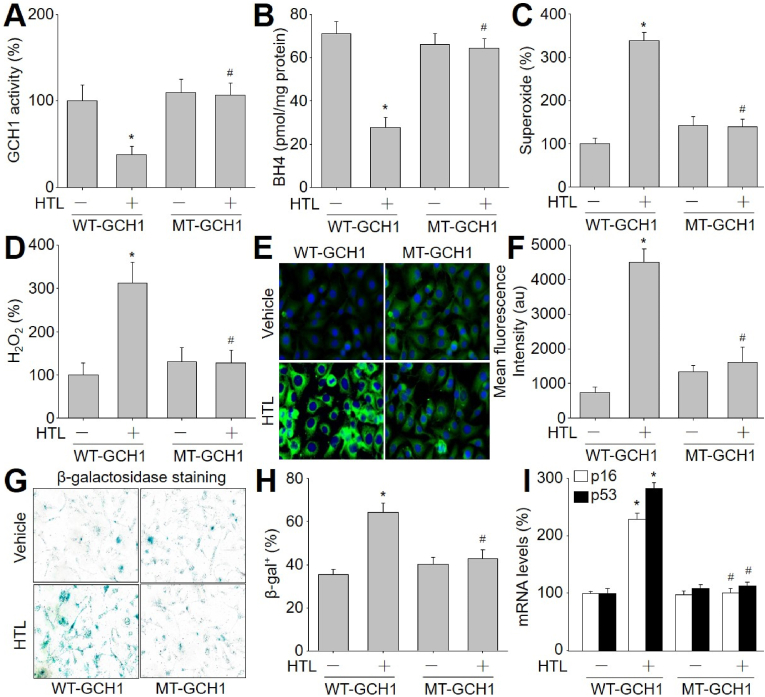
**Enforced expression of S-nitrosylation-resistant GCH1 prevents oxidative stress and cell senescence in endothelial cells treated with homocysteine thiolactone (HTL)**. Human umbilical vein endothelial cells were infected with adenovirus expressing wildtype GCH1 (WT) or mutated GCH1 C141A (MT-GCH1) for 48 h followed by incubation with (HTL (1 mM) for 24 h. (**A**) Total cell lysates were subjected to measure GCH1 activity. (**B**) BH4 content in total cell lysate was determined by HPLC. (**C**) Intracellular superoxide was assayed by DHE fluorescence. (**D**) Intracellular hydrogen peroxide (H_2_O_2_) was assayed by Amplex Red. (**E** and **F**) Representative images of mitochondrial peroxynitrite determined by MitoPY1 in **E** and quantitative analysis in **F** were shown. (**G** and **H**) Representative images of senescence-associated β-galactosidase staining in **G** and quantitative analysis in **H** were shown. (**I**) The gene expressions of p53 and p16 were measured by qPCR. N = 5 per group. *P < 0.05 *vs.* WT-GCH1 alone. ^#^*P* < 0.05 *vs.* WT-GCH1 plus HTL. A one-way ANOVA followed by Tukey's HSD test was used to determine *P* value between two groups in **A, B, C, D**, **F**, **H,** and **I**. (For interpretation of the references to colour in this figure legend, the reader is referred to the Web version of this article.)

To examine whether HTL inhibits GCH1 activity through GCH1 S-nitrosylation at cysteine 141 in cells, we infected HUVECs with adenovirus expressing WT-GCH1 or mutant of GCH1 cysteine 141 to alanine (MT-GCH1), which is S-nitrosylation-resistant. As expected, HTL decreased GCH1 activity and BH4 content (Fig. 3A and B), and increased ROS productions including superoxide (Fig. 3C), hydrogen peroxide (Fig. 3D), and mitochondrial peroxynitrite (Fig. 3E and F) in cells expressing WT-GCH1, but not in cells expressing MT-GCH1, indicating that HTL through GCH1 S-nitrosylation at cysteine 141 induces oxidative stress in endothelial cells.

### 4.5 HTL induces endothelial cell senescence through GCH1 S-nitrosylation

Accumulation of senescent cells in vascular endothelium contributes to vascular stiffness and increases the risk of cardiovascular diseases [32,33]. Thus, we tested the effects of GCH1 S-nitrosylation in endothelial cellular senescence. As presented in Fig. 3G, H, and 3I, HTL promoted cellular senescence determined by β-galactosidase staining and increased stable senescence-associated reference gene expressions of p16 and p53 in HUVECs expressing WT-GCH1, but not in HUVECs expressing MT-GCH1.

### 4.6 HTL-promoted endothelial cell senescence and oxidative stress are prevented by NAC, tempol, and L-sepiapterin in HUVECs

**Fig. 4:**
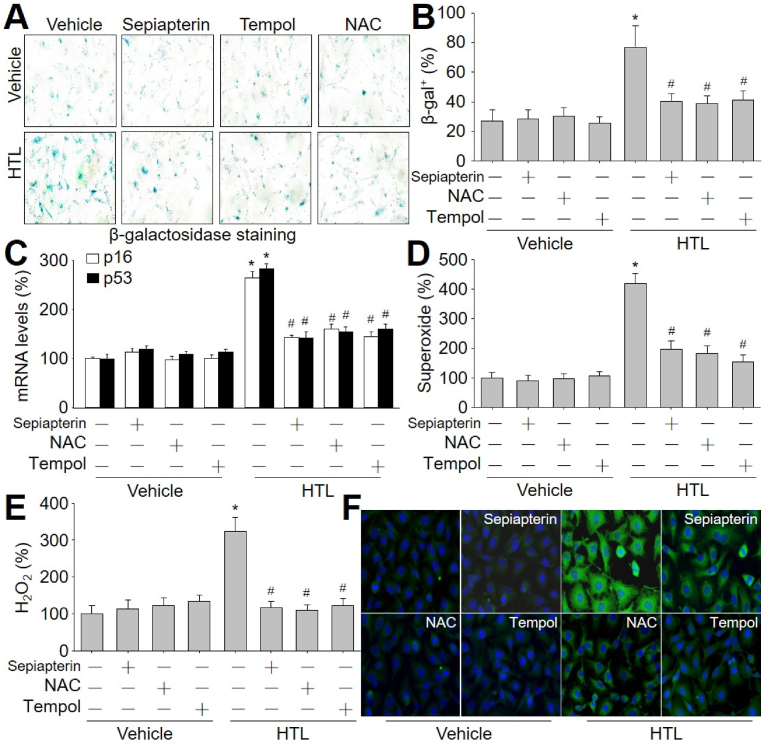
**Homocysteine thiolactone (HTL) promotes cell senescence and oxidative stress in endothelial cells, which are inhibited by N-acetyl-cysteine (NAC), tempol, and sepiapterin.** Human umbilical vein endothelial cells were pretreated with NAC (2.5 mM), L-sepiapterin (10 μM) or tempol (1 mM) for 30 min followed by incubation with HTL (1 mM) for 24 h. (**A** and **B**) Representative images of senescence-associated β-galactosidase staining in **A** and quantitative analysis in **B** were shown. (**C**) The gene expressions of p53 and p16 were measured by qPCR. (**D**) Intracellular superoxide was assayed by DHE fluorescence. (**E**) Intracellular hydrogen peroxide (H_2_O_2_) was assayed by Amplex Red. (**F**) Mitochondrial peroxynitrite was determined by MitoPY1. N = 5 per group. *P < 0.05 *vs.* Vehicle alone. ^#^*P* < 0.05 *vs.* HTL alone. A one-way ANOVA followed by Tukey's HSD test was used to determine *P* value between two groups in **B**, **C**, **D**, **E**, and **F**. (For interpretation of the references to colour in this figure legend, the reader is referred to the Web version of this article.)

Knowing that HTL via dysfunctional GCH1 signaling promotes cell senescence, we proposed that the normalization of GCH1 signaling prevents HTL-induced cell senescence. To this end, pharmacological approaches including NAC, L-sepiapterin at 10 μM as BH4 precursor [34,35], and tempol at 1 mM as ROS scavenger [36], were applied to HTL-treated HUVECs (Online Fig. S1C). In depicted, HTL-induced cell senescence (Fig. 4A–C) and oxidative stress (Fig. 4D–F), liking mitochondrial dysfunction [37], were dramatically reversed by these reagents.

### 4.7 Overexpression of iNOS promotes nitrosative stress, oxidative stress and cell senescence, which is abolished by exogenous expression of MT-GCH1 in HUVECs

**Fig. 5:**
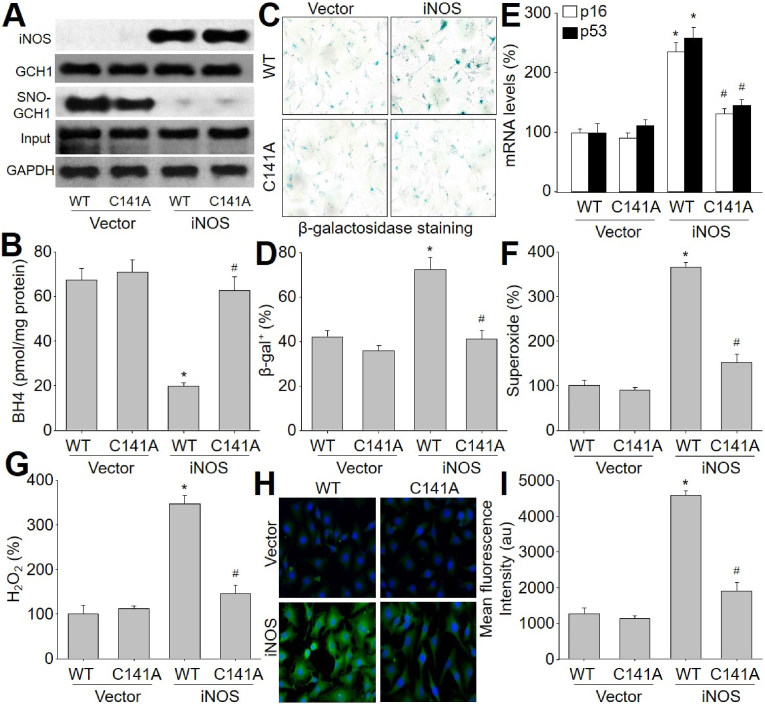
**Enforced expression of S-nitrosylation-resistant GCH1 prevents oxidative stress and cell senescence in iNOS-overexpressed endothelial cells**. Human umbilical vein endothelial cells were infected with adenovirus expressing wildtype GCH1 (*WT*) or mutated GCH1 (C141A) plus iNOS cDNA with for 48 h. (**A**) Total cell lysates were subjected to measure protein levels of iNOS, total GCH1, and S-nitrosylated GCH1. (**B**) BH4 content in total cell lysate was determined by HPLC. (**C** and **D**) Representative images of senescence-associated β-galactosidase staining in **C** and quantitative analysis in **D** were shown. (**E**) The gene expressions of p53 and p16 were measured by qPCR. (**F**) Intracellular superoxide was assayed by DHE fluorescence. (**G**) Intracellular hydrogen peroxide (H_2_O_2_) was assayed by Amplex Red. (**H** and **I**) Representative images of mitochondrial peroxynitrite determined by MitoPY1 in **H** and quantitative analysis in **I** were shown. N = 5 per group. *P < 0.05 *vs. WT* plus vector. ^#^*P* < 0.05 *vs. WT* plus iNOS. A one-way ANOVA followed by Tukey's HSD test was used to determine *P* value between two groups in **B, D, E**, **F**, **G** and **I**. (For interpretation of the references to colour in this figure legend, the reader is referred to the Web version of this article.)

To provide additional evidence between iNOS and GCH1, we infected HUVECs with adenovirus expressing iNOS and MT-GCH1 (C141A). As expected, iNOS overexpression mimicked the effects of HTL to induce GCH1 S-nitrosylation and BH4 inhibition (Fig. 5A and B), cell senescence (Fig. 5C–E), and oxidative stress (Fig. 5F–I), and in HUVECs, compared to vector. However, enforced expression of MT-GCH1 abolished all alternations induced by iNOS overexpression, compared to WT-GCH1. These data solidly indicate that iNOS-mediated nitrosative stress inhibits GCH1 through direct C141 S-nitrosylation.

### 4.8 Administration of HTL damages long-term memory in mice

We have previously established HHcy model by feeding rats with HTL, a physiologic metabolite of homocysteine with high reactivity [38], for 16 weeks. Therefore, we determined the effects of HTL on cognitive functions by feeding mice with HTL for 16 weeks (Online Fig. S2A). The long-term memory was evaluated by step-down test at the 24th after training. As shown in Table 1, compared to saline-treated mice, the latency of step-down test in HTL-treated mice was much shorter and the error number was much higher in HTL-treated mice. HTL-induced impairment of long-term memory was further confirmed by passive avoidance step-through task test, as decreased the latency and increased the error number (Table 1). These data indicate that HTL damages long-term memory in mice.

**Table 1:** Effects of HTL on Step-down and Step-through tests in *WT* and *iNOS*^*−/−*^ mice.

Groups	*WT* (Control)	*iNOS*^*−/−*^	*WT* + HTL	*iNOS*^*−/−*^ + HTL
N	14	10	13	15
Step-down				
Latency	163 ± 18	171 ± 25	65 ± 14^a^	165 ± 19^b^
Number of errors	0.23 ± 0.08	0.29 ± 0.10	2.84 ± 0.22^a^	0.27 ± 0.07^b^

Step-through				
Latency	186 ± 27	172 ± 31	27±8^a^	155 ± 17^b^
Number of errors	0.44 ± 0.06	0.43 ± 0.05	3.59 ± 0.10^a^	0.45 ± 0.04^b^

The experimental protocols were described and graphical in Online Fig. S2A. Data are expressed as means ± SE. ^a^*P* < 0.05 *vs. WT*. ^b^*P* < 0.05 *vs. WT* plus HTL. A one-way ANOVA followed by Scheffe test was used to determine *P* value between two groups in this table.

### 4.9 HTL impairs the functions of learning and spatial memory in mice

**Fig. 6:**
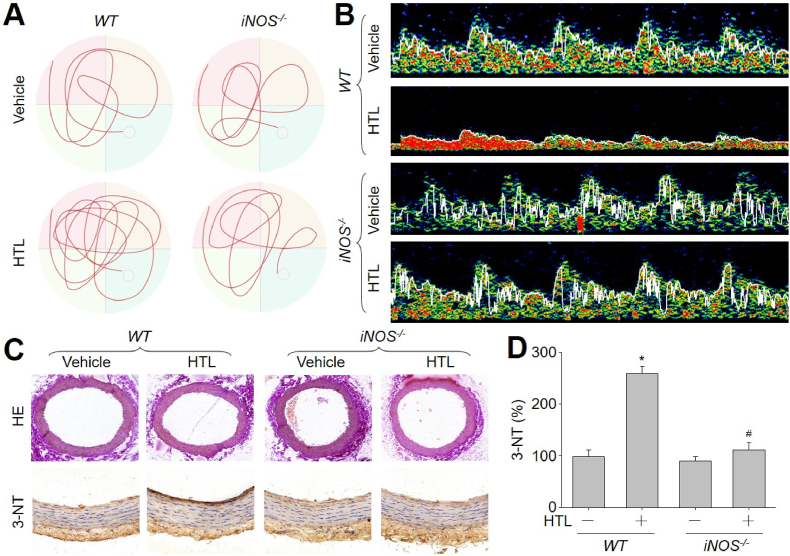
**Gene knockout of iNOS (*iNOS***^***−/−***^**) eliminates the detrimental effects of homocysteine thiolactone (HTL) on cognitive functions and the blood flow of middle cerebral arteries (MCA) in mice**. The experimental protocols were described and graphical in Online Fig. S2A. (**A** and **B**) The representative swimming traces of MWM test in **A** and the TCD pictures in **B** were shown. Quantitative analyses about MWM test, MCA flow velocity, and MCA pulse wave velocity were summarized in Table 2. (**C** and **D**) Left common carotid arteries isolated from wildtype (*WT*) and *iNOS*^*−/−*^ mice were used for IHC analysis of 3-nitrotyrosine (3-NT) and HE staining in **C**. Quantitative analyses of 3-NT were shown in **D**. N = 10–15 per group. *P < 0.05 *vs. WT*. ^#^*P* < 0.05 *vs. WT* plus HTL. A one-way ANOVA followed by Scheffe test was used to determine *P* value between two groups in **D**.

We next performed the MWM test to ascertain the function of learning and spatial memory in mice. As presented in Fig. 6A and Table 2, swimming distance in quadrant, ratio of swimming distance in quadrant to total swimming distance, swimming time in quadrant, ratio of swimming time in quadrant to total swimming time, and number of crossing platform were decreased, compared to saline-treated mice. Conversely, swimming distance out of quadrant, swimming time out of quadrant, and the latency were increased in HTL-fed mice. By analyzing the swimming distance, swimming speed in quadrant, swimming speed out quadrant, and the average of swimming speed (Table 2), we found there was no significant difference between any two groups, demonstrating that motor function was not impaired by HTL treatment. Getting these data together, it suggests that HTL impairs learning and spatial memory in mice.

**Table 2:** Effects of HTL on cognitive functions and MCA flow velocity in *WT* and *iNOS*^*−/−*^ mice.

Groups	*WT* (Control)	*iNOS*^*−/−*^	*WT* + HTL	*iNOS*^*−/−*^+ HTL
Swimming distance in quadrant (m)	2.69 ± 0.31	2.57 ± 0.39	1.27 ± 0.33^a^	2.47 ± 0.35^b^
Swimming distance out of quadrant (m)	3.49 ± 0.58	3.81 ± 0.52	4.85 ± 0.47^a^	3.53 ± 0.46^b^
Total swimming distance (m)	6.13 ± 0.78	6.49 ± 0.91	6.09 ± 0.62	6.17 ± 0.83
Swimming distance in quadrant/Swimming distance (Ratio)	0.44 ± 0.04	0.43 ± 0.05	0.21 ± 0.03^a^	0.40 ± 0.03^b^
Swimming time in quadrant (s)	20.36 ± 4.58	21.07 ± 4.11	13.59 ± 3.09^a^	20.91 ± 4.07^b^
Swimming time out of quadrant (s)	26.41 ± 4.17	28.29 ± 5.31	31.90 ± 6.78^a^	27.15 ± 5.57^b^
Swimming time in quadrant/Total swimming time (Ratio)	0.44 ± 0.06	0.43 ± 0.05	0.30 ± 0.03^a^	0.45 ± 0.04^b^
Latency (s)	19 ± 2.33	22 ± 2.58	35 ± 3.89^a^	25 ± 2.66^b^
Number of crossing platform	9.72 ± 1.37	10.13 ± 1.56	5.27 ± 1.42^a^	8.89 ± 0.99^b^
Swimming speed in quadrant (mm/s)	132.12 ± 21.57	121.97 ± 19.46	108.64 ± 13.51	118.27 ± 15.60
Swimming speed out quadrant (mm/s)	135.46 ± 14.47	134.68 ± 16.94	142.04 ± 15.48	130.02 ± 16.02
Average Swimming speed (mm/s)	133.26 ± 16.95	132.45 ± 19.83	135.33 ± 13.33	128.54 ± 16.46
MCA V_S_ (cm/s)	35.17 ± 4.29	36.09 ± 3.81	26.27 ± 3.89^a^	33.14 ± 4.74^b^
MCA V_d_ (cm/s)	21.07 ± 3.61	24.01 ± 3.74	13.75 ± 2.36^a^	22.96 ± 3.59^b^
PWV (cm/s)	275 ± 36	239 ± 31	749 ± 41^a^	328 ± 39^b^

The experimental protocols were described and graphical in Online Fig. S2A. The original recordings were presented Fig. 6A. Data are expressed as means ± SE. ^a^*P* < 0.05 *vs. WT*. ^b^*P* < 0.05 *vs. WT* plus HTL. A one-way ANOVA followed by Scheffe test was used to determine *P* value between two groups in this table.

### 4.10 Long-term treatment of HTL reduces cerebrovascular blood flow in mice

Hypoperfusion of cerebral blood vessels could contribute to the neuronal dysfunctions and cognitive impairments [39]. Thus, we measured the cerebrovascular functions in HTL-fed mice. As shown in Fig. 6B and Table 1, the flow velocity of middle cerebral arteries (MCA) was dramatically reduced after 16-week administration of HTL in mice, as indexed by the decreased systolic MCA flow velocity (Vs) and diastolic MCA flow velocity (Vd), compared to saline-treated mice. Further, MCA pulse wave velocity (PWV) dramatically increased after 16-week administration of HTL, indicating that HTL induced MAC stiffness.

### 4.11 HTL via iNOS induces vascular stiffness and cognitive declines in mice

We next tested the role of iNOS in HTL-induced vascular cognitive dysfunctions. As depicted in Fig. 6A and B, Table 1, Table 2, after long-term HTL-administration, *WT* mice displayed significantly accelerated vascular stiffness, impaired vascular cognitive functions and decreased MCA flow, compared with those of *iNOS*^*−/−*^ mice, suggesting that iNOS is required for HTL-induced VCI *in vivo*.

### 4.12 The iNOS is crucial for HTL-induced vascular nitrosative stress, GCH1 signaling inhibition, and oxidative stress in mice

As depicted in Fig. 6C and D, Online Figure S2B, S2C and S2D, and Online Figs. S3A–S3D, long-term HTL administration increased NO production and GCH1 S-nitrosylation, reduced GCH1 activity and BH4 content, and induced *in vivo* oxidative damage in *WT* mice, but not in *iNOS*^*−/−*^ mice.

Besides, both methylation and transsulfuration are involved in HHcy-induced cell dysfunction [[40], [41], [42], [43]]. We next checked methylation and transsulfuration *in vivo*. As shown in Online Figs. S4A–S4D, long-term treatment of HTL dramatically increased plasma levels of HTL and slightly increased plasma homocysteine in both *WT* mice and *iNOS*^*−/−*^ mice. While, cystathionine beta synthase activity and protein mono-methylation were comparable in all groups. These data reveal that S-nitrosylation is essential for HTL-induced VCI, rather than methylation and transsulfuration.

### 4.13 Exogenous expression of S-nitrosylation-resistant GCH1 abolishes HTL-induced cognitive impairments in mice

**Fig. 7:**
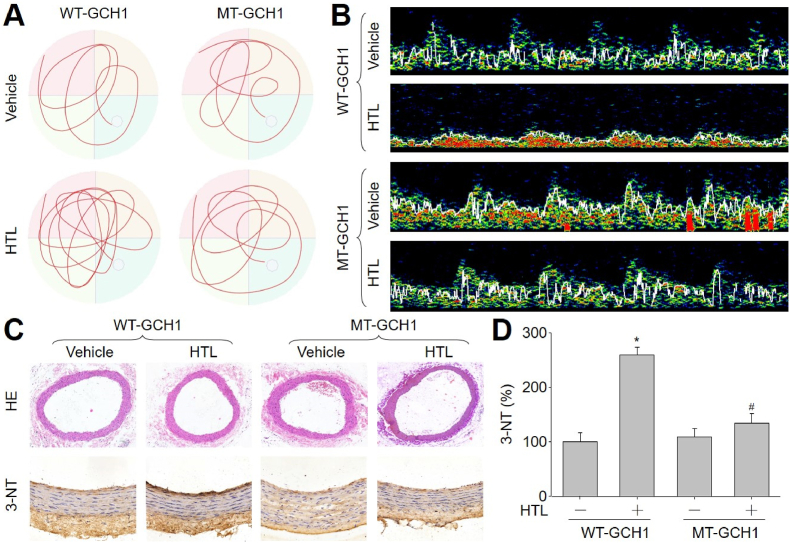
**Cognitive impairments are rescued by exogenous expression of GCH1 C141A mutant (MT-GCH1) in mice fed with homocysteine thiolactone (HTL)**. The experimental protocols were described and graphical in Online Fig. S5A. (**A** and **B**) Before sacrificed, MWM test was performed to determine spatial learning and memory in **A**. MCA flow velocity was determined by TCD in **B**. Quantitative analyses about MWM test, MCA flow velocity, and MCA pulse wave velocity were summarized in Table 4. (**C** and **D**) Left common carotid arteries isolated from mice were used for IHC analysis of 3-nitrotyrosine (3-NT) and HE staining in **C**. Quantitative analyses of 3-NT were shown in **D**. N = 10–15 per group. *P < 0.05 *vs.* WT-GCH1. ^#^*P* < 0.05 *vs.* WT-GCH1 plus HTL. A one-way ANOVA followed by Scheffe test was used to determine *P* value between two groups in **D**.

We finally examined the role of GCH1 S-nitrosylation in HTL-induced VCI *in vivo* by infecting mice with adeno-associated virus 9 (AAV9) expressing WT-GCH1 or MT-GCH1 (Online Fig. S5A). As indicated in Fig. 7A, Table 3, and Table 4, HTL significantly induced multiple deficits of learning and memory in mice infected with AAV9 expressing WT-GCH1, but not in mice infected with AAV9 harboring MT-GCH1, demonstrating that HHcy via GCH1 S-nitrosylation at C141 induces cognitive impairments *in vivo*.

**Table 3:** Effects of exogenous MT-GCH1 (C141A) on Step-down and Step-through tests in HTL-fed mice.

Groups	WT-GCH1 (Control)	MT-GCH1	WT-GCH1 +HTL	MT-GCH1 +HTL
N	10	13	12	15
Step-down				
Latency	178 ± 21	169 ± 25	57 ± 14^a^	159 ± 26^b^
Number of errors	0.83 ± 0.12	0.90 ± 0.17	3.81 ± 0.39^a^	0.98 ± 0.15^b^

Step-through				
Latency	186 ± 27	161 ± 19	29±8^a^	157 ± 14^b^
Number of errors	0.47 ± 0.12	0.49 ± 0.11	2.25 ± 0.08^a^	0.58 ± 0.13^b^

The experimental protocols were described and graphical in Online Fig. S5A. Data are expressed as means ± SE. ^a^*P* < 0.05 *vs.* WT-GCH1. ^b^*P* < 0.05 *vs.* WT-GCH1 plus HTL. A one-way ANOVA followed by Scheffe test was used to determine *P* value between two groups in this table.

**Table 4:** Effects of exogenous MT-GCH1 (C141A) on cognitive functions and MCA flow velocity in HTL-fed mice.

Groups	WT-GCH1	MT-GCH1	WT-GCH1+HTL	MT-GCH1+HTL
Swimming distance in quadrant (m)	2.87 ± 0.34	2.85 ± 0.52	1.39 ± 0.37^a^	2.61 ± 0.42^b^
Swimming distance out of quadrant (m)	2.98 ± 0.51	2.99 ± 0.55	4.57 ± 0.54^a^	2.91 ± 0.46^b^
Total swimming distance (m)	5.83 ± 0.82	5.90 ± 0.91	5.81 ± 0.89	5.79 ± 0.77
Swimming distance in quadrant/Swimming distance (Ratio)	0.49 ± 0.04	0.47 ± 0.07	0.24 ± 0.03^a^	0.43 ± 0.05^b^
Swimming time in quadrant (s)	23.16 ± 3.41	24.19 ± 3.01	12.53 ± 2.94^a^	22.74 ± 3.29^b^
Swimming time out of quadrant (s)	24.86 ± 4.07	25.61 ± 4.64	39.97 ± 5.09^a^	24.15 ± 3.91^b^
Swimming time in quadrant/Total swimming time (Ratio)	0.47 ± 0.12	0.49 ± 0.11	0.25 ± 0.08^a^	0.49 ± 0.13^b^
Latency (s)	22 ± 2.65	23 ± 2.88	37 ± 4.12^a^	27 ± 3.01^b^
Number of crossing platform	12.17 ± 1.49	13.01 ± 1.52	7.93 ± 0.93^a^	10.29 ± 1.17^b^
Swimming speed in quadrant (mm/s)	123.92 ± 26.77	117.81 ± 27.71	110.93 ± 18.45	114.78 ± 23.66
Swimming speed out quadrant (mm/s)	119.87 ± 23.31	116.75 ± 25.53	114.33 ± 23.68	120.49 ± 19.93
Average Swimming speed (mm/s)	121.49 ± 23.33	120.41 ± 26.53	111.73 ± 30.76	123.19 ± 26.17
MCA V_S_ (cm/s)	37.48 ± 5.16	35.67 ± 4.89	24.73 ± 4.25^a^	34.57 ± 4.91^b^
MCA V_d_ (cm/s)	23.29 ± 3.14	22.58 ± 3.70	14.71 ± 2.96^a^	20.36 ± 3.43^b^
PWV (cm/s)	249 ± 26	264 ± 30	705 ± 51^a^	381 ± 34^b^

The experimental protocols were described and graphical in Online Fig. S5A. The original recordings were presented Fig. 7A. Data are expressed as means ± SE. ^a^*P* < 0.05 *vs.* WT-GCH1. ^b^*P* < 0.05 *vs.* WT-GCH1 plus HTL. A one-way ANOVA followed by Scheffe test was used to determine *P* value between two groups in this table.

### 4.14 GCH1 S-nitrosylation is required for HTL-induced cerebral vascular dysfunctions and oxidative stress in mice

We also examined the role of GCH1 S-nitrosylation in HTL-induced cerebrovascular dysfunctions *in vivo*. As shown in Fig. 7B and Table 4, HTL induced cerebrovascular dysfunctions including reduced MCA blood flow and increased MCA PWV in mice infected with AAV9 expressing WT-GCH1, but not in mice infected with AAV9 harboring MT-GCH1. Further, nitrosative stress (Fig. 7C and D) and *in vivo* oxidative damage (Online Figs. S5B–S5D) were increased in HTL-treated mice, but not in mice infected with AAV9 harboring MT-GCH1.

### 4.15 Increases GCH1 S-nitrosylation and impaired cognitive functions in patients with HHcy

To provide translational perspectives of this study, we conducted a pilot experiment by collecting bloods from HHcy patients to determine the association between GCH1 S-nitrosylation and cognitive functions. As presented in Online Figs. S6A and S6B, the cerebral blood flow, as determined by computed tomography perfusion imaging, was dramatically decreased in patients with HHcy, compared to patients without HHcy. MMSE score was also lower in HHcy patients (Online Fig. S6C). Further, increased GCH1 S-nitrosylation and reduced GCH1 activity were detected in white blood cells isolated from HHcy patients (Online Figs. S6D and S6E). Also, *in vivo* oxidative damage, as increased F_2_-isoprostanes in urine and blood, was observed in HHcy patients (Online Figs. S6F and S6G). Although the pilot experiment did not establish the cause-effect association between GCH1 S-nitrosylation and cognitive functions, it still implies the important role of GCH1 S-nitrosylation in HHcy-instigated VCI clinically.

## 5 Discussion

In the present study, we provide the first evidence that HHcy induces VCI *in vivo* through GCH1 S-nitrosylation. Using multiple approaches in the model of HTL-induced VCI and human samples from patients with HHcy, we uncovered evidence that implicates iNOS-mediated nitrosative stress and suppression of the GCH1-BH4-eNOS axis in oxidative stress. Indeed, our findings suggest that metabolic inflammation and its master mediator, such as iNOS, are critical elements of the pathophysiology of VCI. This finding is highly important not only because we unveil a previously unrecognized pathophysiological mechanism of VCI through GCH1 S-nitrosylation, but also explain how nitrosative stress links to oxidative stress during the process of endothelial cell senescence.

The major finding of this study is that GCH1 activity is regulated by S-nitrosylation. Protein post-translational modifications, including phosphorylation, ubiquitination, glycation, etc., are important to determine protein functions and play key roles in many cellular processes [44]. This study provides further evidence to support the proposal that GCH1 function is tightly controlled by GCH1 S-nitrosylation. First, human recombinant GCH1 protein is S-nitrosylated by SNP in a concentration-dependent manner. Second, GCH1 S-nitrosylation is detectable in HTL-treated cells and animals using biotin-switch method. Further, we identified that the S-nitrosylation site in GCH1 protein is cysteine 141 in human protein but not in cysteine 18 and 212 because only mutation of cysteine 141 to alanine abolished GCH1 protein S-nitrosylation. To best of our knowledge, it is the first study to report that GCH1 activity is regulated by S-nitrosylation.

Another notable finding is that GCH1 S-nitrosylation contributes to endothelial cell senescence and HTL-instigated VCI. This conclusion is supported by several observations. First, inhibition of protein S-nitrosylation by NAC or L-NIL reversed HTL-induced GCH1 inhibition *in vitro*. Second, gene knockout of iNOS inhibited HTL-induced GCH1 S-nitrosylation and endothelial cell senescence. Third, the formation of VCI following HTL administration was reduced in *iNOS*^*−/−*^ mice, determined by step-down avoidance test, passive avoidance step-through task test, and MWM test (Fig. 6A, Table 1, Table 2). We further demonstrated that enforced expression of mutated GCH1 at cysteine 141 to alanine, which is resistant to S-nitrosylation, has a marked protective effect against cognitive dysfunctions by behavior tests (Fig. 7A, Table 3, Table 4). These findings suggest a critical role for GCH1 S-nitrosylation in endothelial cell senescence and VCI.

It has been reported that the methylation/transsulfuration pathway is involved in homocysteine irreversible consumption [[40], [41], [42], [43]]. However, we did not observe long-term HTL administration significantly affects methylation or transsulfuration in mice (Online Figs. S4C and S4D). We reasoned that the method used to induce HHcy might produce this discrepancy. As a physiologic metabolite of homocysteine, HTL is more cytotoxic than homocysteine [45]. Using this biological character, we can mimick the cytotoxic effects of HHcy by feeding animals with HTL and successfully replicated the preclinical model of HHcy in mice, but not a real HHcy. In this model, plasma homocysteine is increased in HTL-treated mice, but not as highly as HTL (Online Figs. S4A and S4B). In fact, it is very difficult to induce HHcy model except genetic manipulations. For examples, genetic manipulation of either cystathionine beta synthase or methylenetetrahydrofolate reductase can produce mouse models of HHcy, showing thickened cerebral arteriolar walls and blood brain barrier dysfunction [46]. Of course, whether HHcy, rather than HTL, inhibits GCH1 activity through methylation or transsulfuration need further investigations.

Some limitations of this study should be considered. As such, aging-induced functional and structural alterations of the microcirculation contribute to the pathogenesis of range of age-related diseases, including vascular cognitive impairment, Alzheimer disease, sarcopenia, and kidney and eye disease [47]. Emerging studies highlight that perturbation of endothelial cell senescence is a potential link between inflammation and aging [5,48]. In this study, we have clearly demonstrated the HTL-induced iNOS-mediated GCH1 S-nitrosylation promotes endothelial cell senescence, which is identified by stable senescence-associated reference genes combined with established markers by beta-gal staining as referred by Alejandra Hernandez Segura and José Antonio de Mera-Rodríguez [49,50]. We are not convinced that endothelial cell senescence is critical to vascular ageing because it is hard to detect endothelial cellular senescence in aged vascular wall. Anyway, we have provided experimental evidence to demonstrate the role of endothelial cell senescence in MCA stiffness. Another limitation is that the specifics of how the behavioral tests are conducted can affect the results. Our conclusion is only based on the observations obtained from the methods used in this study. Further study is warranted to confirm these findings by other methods.

**Fig. 8:**
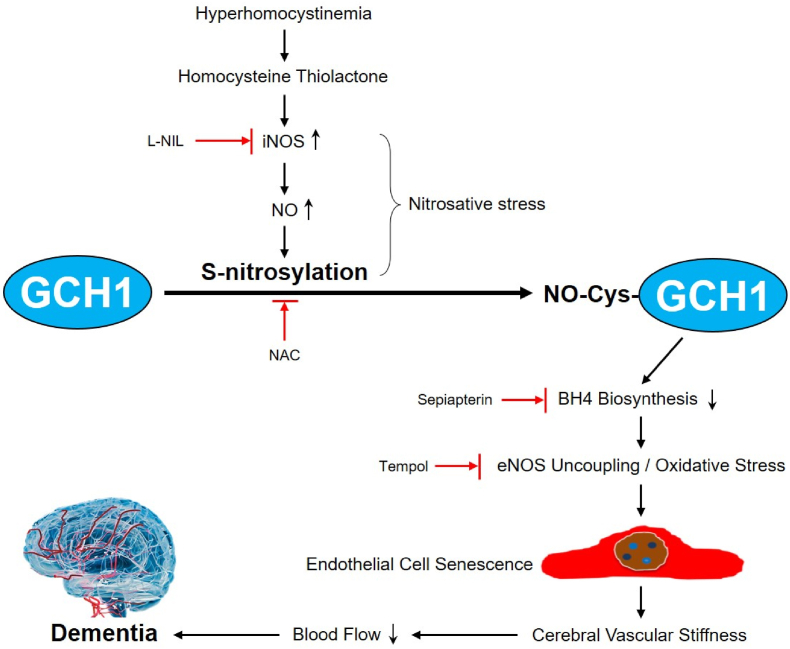
**Proposed mechanism of hyperhomocystinemia-induced VCI.** Under hyperhomocystinemia, homocysteine thiolactone, as a major metabolite of homocysteine, induces iNOS positively expressed in cerebrovascular endothelial cells to produce aberrant NO to trigger nitrosative stress. NO S-nitrosylates GCH1 protein at cysteine 141 to inhibit its activity, resulting in BH4 deficiency. Under this condition, eNOS is uncoupled to produce reactive oxygen species (ROS). Excessive ROS productions induce oxidative stress to promote endothelial cell senescence and consequent cerebrovascular stiffness. Therefore, blood supply to brain is decreased, and cognitive functions are impaired. In perspective, pharmacological approaches, e.g. protein S-nitrosylation by N-acetyl-cysteine (NAC), iNOS selective inhibitor L-N6-1-Iminoethyl-lysine (L-NIL), BH4 supplementation by BH4 precursor L-sepiapterin, and ROS scavenger tempol, prevent cerebral vascular dysfunction, increase brain blood supply, and improve the cognitive functions.

In conclusion, HHcy upregulates iNOS signaling to instigate GCH1 S-nitrosylation, leading to GCH1 suppression and subsequent endothelial cell senescence (Fig. 8). In this way, HHcy induces cerebrovascular dysfunctions and the impairments of learning and memory. In perspective, pharmacological approaches, e.g. S-nitrosylation blockage, iNOS selective inhibition, BH4 supplementation, and ROS clearance, might prevent VCI in patients with HHcy.

## Author contributions

Y.L.Y. conceived the study, performed most experiments, and wrote the draft of the paper. Y.C., F.R., L.W., M.L.Z. J.X.L., Q.Q.W., C.B.L., C.L., and Y.P.B. partially conducted some experiments. S.X.W. and J.Z.W. helped to analyze the data and organize the manuscript. P.L. revised this paper and supervised the study.

## Declaration of competing interest

We all authors stated that we have no conflicts of interest.Ya-Ling Yin, Yuan Chen, Feng Ren, Lu Wang, Mo–Li Zhu, Jun-Xiu Lu, Qian-Qian Wang, Cheng-Biao Lu, Chao Liu, Yong-Ying Bai, Shuang-Xi Wang, Jian-Zhi WangPeng Li.

## Data Availability

Data will be made available on request.
