# Supplementary material for: Nitrosative stress induced by homocysteine thiolactone drives vascular cognitive impairments via GTP cyclohydrolase 1 S-nitrosylation in vivo
Source: Redox Biol. 2022 Nov 13;58:102540. doi: 10.1016/j.redox.2022.102540 (PMC9673109; doi:10.1016/j.redox.2022.102540)
Supplement: Multimedia component 1 [file mmc1.docx]

**Online Supplements**

**Nitrosative stress induced by homocysteine thiolactone drives vascular cognitive impairments via GTP cyclohydrolase 1 S-nitrosylation *in vivo***

Ya-Ling Yin^1^, Yuan Chen^2^, Feng Ren^1^, Lu Wang^1^, Mo-Li Zhu^3^, Jun-Xiu Lu^1,3^, Qian-Qian Wang^3^, Cheng-Biao Lu^1^, Chao Liu^4^, Yong-Ying Bai^3,5^, Shuang-Xi Wang^2,3,4,#^, Jian-Zhi Wang^6,#^, Peng Li^1,3,#^

^1^Sino-UK Joint Laboratory of Brain Function and Injury and Department of Physiology and Neurobiology, School of Basic Medical Sciences, Xinxiang Medical University, Xinxiang, China; ^2^The Key Laboratory of Cardiovascular Remodeling and Function Research, Chinese Ministry of Education, Chinese National Health Commission and Chinese Academy of Medical Sciences, The State and Shandong Province Joint Key Laboratory of Translational Cardiovascular Medicine, Qilu Hospital of Shandong University, Jinan, Shandong, China; ^3^School of Pharmacy, Henan International Joint Laboratory of Cardiovascular Remodeling and Drug Intervention, Xinxiang Medical University, Xinxiang, Henan, China; ^4^Hubei Key Laboratory of Cardiovascular, Cerebrovascular, and Metabolic Disorders, Hubei University of Science and Technology, Xianning, China; ^5^Department of Geriatric Medicine and Department of Cardiovascular Medicine, Coronary Circulation Center, National Clinical Research Center for Geriatric Disorders, Xiangya Hospital, Central South University, Changsha, Hunan, China; ^6^Department of Pathophysiology, School of Basic Medicine and the Collaborative Innovation Center for Brain Science, Key Laboratory of the Ministry of Education of China for Neurological Disorders, Tongji Medical College, Huazhong University of Science and Technology, Wuhan, Hubei, China

^#^Correspondence to Shuang-Xi Wang, Jian-Zhi Wang, and Peng Li, No 601, Jinsui Avenue, Xinxiang City, Henan Province, China 453003. Email: shuangxiwang@sdu.edu.cn, [wangjz@mail.hust.edu.cn](mailto:wangjz@mail.hust.edu.cn), and pengli@xxmu.edu.cn

**Materials and methods**

**Materials**

Sodium nitroprusside (SNP), homocysteine thiolactone (HTL), L-N6-1-Iminoethyl-lysine (L-NIL), L-sepiapterin, N-acetyl-cysteine (NAC), and tempol were purchased from Sigma-Aldrich Company (Merck KGaA, Darmstadt, Germany). Primary antibodies against inducible NO synthase (iNOS), GTP cyclohydrolase 1 (GCH1), 3-nitrotyrosine (3-NT), mono-methylation and GAPDH were purchased from Cell Signaling Transduction Company. Protein A/G plus-agarose and secondary antibody were obtained from Santa Cruz Biotechnology Inc. (Santa Cruz, CA). Cellular senescence assay kit was bought from Cell Biolabs (San Diego, CA, USA). Recombinant human GCH1 protein and biotin switch assay kit (S-Nitrosylation) (ab236207) were obtained from Abcam Company (San Francisco, USA). Dihydroethidium (DHE) was purchased from Calbiochemical Company (USA). Amplex red was purchased from Invitrogen. MitoPY1 was bought from Tocris. 8iPF2α-d4 was obtained from Cayman chemical, Ann Arbor, MI, USA. All drug concentrations are expressed as final working concentrations in the buffer.

**Animals**

Male wild type (*WT*) C57BL6 mice and iNOS gene knockout (*iNOS^-/-^*) mice, obtained from Jackson Laboratories (Bar Harbor, ME), at 4-6 weeks of age with 20-25 g body weight were used in this study. All animals were housed individually in cages at a room temperature of 18-22 °C with a 12-h light/dark cycle and given free access to food and water. The environment, such as cleanness, humidity, ventilation, air flow speed, air pressure, and automatic timer, was strictly control. This study was carried out in accordance with the ethical standards laid down in the 1964 Declaration of Helsinki and its later amendments. The animal protocol was reviewed and approved by the Animal Care and Use Committee, University of Xinxiang Medical University.

**Determinations of flow velocity and pulse wave velocity (PWV) in middle cerebral artery (MCA) by transcranial Doppler (TCD)**

The MCA flow velocity (MCAv) and PWV were measured through the temporal window using 2-MHz pulsed X4-RC TCD (DWL Elctronische Systeme GmbH, Germany) as described previously[1, 2]. Animals were fixed in the normal position and the bilateral temporal parts were sheared. The probe was fixed and held in place using a headband. Under the conditions of power = 6-8 mW/cm^2^, sampling volume = 6 mm^3^, gain = 60-80%, high-pass filter = 50-100 Hz, depth of exploration = 5.5-6.5 mm, the MCA is detectable. The MCA signals were confirmed by carotid compression and neurovascular coupling tests. The systolic MCAv (Vs) and diastolic MCAv (Vd) were recorded. For the measurement of PWV, point A and point B from the anterior wall of MCA were undertaken by the probe. The distance (D) between point A and point B were measured. The electron-machinery time (EMT) and heart rate (HR) were recorded. Both EMT and HR were measured for three times. The average value was used to calculate PWV using this following formula: PWV = D/[(EMTa-EMTb)XHR]. The values of systolic MCAv (Vs), diastolic MCAv (Vd), and PWV were calculated by this machine automatically and exported as a report. The original TCD picture was saved in computer.

**Step-down avoidance test**

Mice were trained in a step-down avoidance test on the tenth day after each stress exposure. Mice were placed on a 7X25X2.5 cm platform. The platform faced a 42X25 cm grid of parallel stainless steel bars that were 0.1 cm in caliber and spaced 1 cm apart. In the training sessions, the animals received a 0.5 mA scramble foot shock for 2 s immediately upon stepping down. The interval of time that elapsed until the mice stepping down and placing all four paws on the grid was defined as the latency. Mice were excluded from the experiment if the waiting time is more than 300 s on the platform during the training in step-down avoidance test. Because we used a single trail based on the animal response, but not multiple trails with a 10-s interval[3-5], it is appropriate to measure long-term memory at the 24^th^ hour after training. The single trail was performed under the conditions of electric current = 0.1-0.5 mA, frequency = 1 Hz, and time of duration = 2-15 s. Then, the mouse was again placed on a platform the latency and number of errors the mice stepped down the platform and were shocked were recorded.

**Passive avoidance step-through task test**

This procedure was carried out as previously described, with some modifications[6]. The apparatus consisted of a light compartment illuminated with a lamp (60 W positioned above the apparatus) and a dark compartment (20X20X40 cm) with an electrifiable grid floor. The two compartments were separated by a black partition with a rectangular doorway (8X8 cm). The floor was constructed of stainless-steel grids 0.2 cm in diameter and at 0.8 cm intervals. Intermittent electric shock (50 Hz, 10 s, 0.5 mA intensity) was delivered to the grid floor of the dark compartment by an isolated stimulator.

An acquisition trial was performed for mice to habituate in both compartments freely for 3 min. During training, each mouse was placed in light compartment, Once the mouse crossed with all four paws into the dark compartment, the door was closed and foot shock was administered. Three minutes later, the mouse was removed from the apparatus and returned to its cage. The mouse waited for more than 100 s to cross to the dark compartment were excluded from the experiment. Twenty-four hours after training, the mouse was again placed in light compartment, the latency to enter the dark compartment with all four paws and number of errors cross into the dark compartment were timed. In the test phase, the latency of mice that did not cross the door was identified as 300 s.

**Morris water maze (MWM) test**

Spatial learning and memory were tested by MWM as we described previously[7-9]. The widely used protocols of MWM test are 1 training per day lasting 6 consecutive days and 2 trainings per day lasting 3 consecutive days. Others liking 1 training per day lasting 4 consecutive days and 1 training per day lasting 5 consecutive days are also used. The threshold of the training is that mice are able to find the platform correctly. We chose 1 training per day lasting 6 consecutive days. During the training phase, one week before the end of the experiments, spatial learning and memory were assessed in an MWM (150 cm in diameter, 50 cm-high) filled with white water (22°C) and surrounded with distal extramaze cues. Before being trained, animals were handled for 1 min a day for 2 days. Mice were then familiarized with water and swimming during two familiarization days (day 1 and day 2) where they had to find a visible platform in the center of a small pool (60 cm diameter) surrounded with curtains (three consecutive trials a day; 60 s-cut-off). On day 0, to evaluate visuomotor deficits, mice were given six trials (90 s-cut-off) to find a visible platform pointed out with a cue in the Morris water maze that was surrounded with white curtains. During the training sessions (days 1-4), animals were required to locate the submerged platform by using distal extramaze cues. They were trained for six trials a day (90 s-cut-off) with an intertrial interval of 5 min for four consecutive days. In order to facilitate spatial learning, mice were introduced from four different starting points, in a randomized daily order. The swimming path and the time used to find the platform were recorded by a video camera fixed on the ceiling of the room, 1.5 m from the water surface. During the probe test, the platform was removed from the pool and spatial memory was evaluated for 60 s. The latency that is the time spent by mice first reaching the location of platform, the percentage of time spent in the target quadrant and the number of platform crossings were recorded.

**Animals and experimental protocols**

In the first part of the animal study (Online Figure S2A), *WT* mice and *iNOS^-/-^* mice received HTL (100 mg/kg/day, 1 ml/kg per two days) administration for 16 consecutive weeks intragastrically. Before sacrifice, MCA flow velocity and PWV were measured by TCD. The cognitive functions were assessed by step-down avoidance test, passive avoidance step-through task test, and MWM test.

In the second part of the animal study (Online Figure S5A), *WT* mice were infected with adeno-associated virus 9 (AAV9) expressing WT-GCH1 or MT-GCH1 (C141A) via tail vein injection followed by 16-week HTL administration (100 mg/kg/day, 1 ml/kg per two days). An injection of AAV9 was repeated once 4 weeks. Before sacrifice, MCA flow velocity and PWV were measured by TCD. The cognitive functions were assessed by step-down avoidance test, passive avoidance step-through task test, and MWM test.

**Hematoxylin-eosin (HE) staining**

As described previously[10], slides with section were placed in a metal staining rack and immersed in the filtered Harris Hematoxylin for 10 seconds. Then, the sections were incubated in EOSIN stain for 30 seconds. Dehydration was performed in ascending alcohol solutions (50%, 70%, 80%, 95% X 2, 100% X 2) followed by clearance with xylene (3-4 X) in in Columbia staining jars. The slides were mount using Permount (xylene based).

**Protein S-nitrosylation assay**

As described previously[11], proteins were extracted according to the manufacturer's specification S-Nitrosylated Protein Detection Assay Kit (Cayman, USA) which is based on the “Biotin-switch” method. Using this method, protein free thiols were blocked with a blocking agent, and then any S-Nitrosothiols in samples were reduced to yield free thiol(s), and subsequently covalently labeled with biotin. After the quantification of protein concentration, equal amounts proteins of control and flavone-treated cells were purified by streptavidin magnetic beads. After purification, streptavidin magnetic beads were washed by water (PH 6.7) for six times, and then S-nitrosylated proteins were separated from streptavidin magnetic beads by 1:5 loading buffer. The S-nitrosylated proteins were detected by western blot.

**Cell cultures**

Human umbilical vein endothelial cells (HUVECs) were obtained from Clonetics Inc. (Walkersville, MD, USA). Cells were grown in endothelial basal medium supplemented with 2% fetal bovine serum (FBS) and penicillin (100 u/ml), and streptomycin (100 µg/ml). All cells were incubated in a humidified atmosphere of 5% CO_2_ + 95% air at 37°C. When 70-80% confluent, the cells were treated with different agents.

Primary murine aortic endothelial cells isolated from mice were grown in Smooth Muscle Cell Medium (Sciencell, USA) supplemented with 2% fetal bovine serum, penicillin (100U/ml) and streptomycin (10mg/ml). In all experiments, cells were used between passages 4 and 8. All cells were incubated at 37°C in a humidified atmosphere of 5% CO_2_ and 95% air. Cells were grown to 80% confluency before being treated with different agents.

For HEK293 cells, cells were cultured in M200 medium supplemented with 2% fetal bovine serum and penicillin (100 u/ml), and streptomycin (100 µg/ml).

**Senescence-associated β-galactosidase staining**

As described previously[12], cells were fixed with 4% paraformaldehyde for 15 min. After fixation, acidic β-galactosidase (β-gal) was assayed in senescence assay buffer (1 mg/mL 5-bromo-4-chloro-3-indolyl β D-galactopyranoside (X-gal), 5 mM K_3_Fe(CN)_6_, 5 mM K_4_Fe(CN)_6_, 2 mM MgCl_2_, 150 mM NaCl, 40 mM citric acid, and 40 mM Na_2_HPO_4_ at pH 6.0) in the dark at 37°C for 16 h. β-gal activity was detected based on SA-β-gal-hydrolyzed X-gal, which produces a blue color.

**Generations of AAV9**

AAV9 construction compassing WT-GCH1 or MT-GCH1 cDNA (AAV9-TIE-cDNA) was generated by according to the manufacturers’ recommendations from Shanghai Genechem Co., Ltd. (Shanghai, China). The endothelial cell specific promoter is “pAAV-TIEp-EGFP-MCS-3Flag-SV40 PolyA”. Viruses were packaged and amplified in HEK293A cells and purified using CsCl_2_ banding followed by dialysis against 10 mM Tris-buffered saline with 10% glycerol. Titering was performed on HEK293 cells using the adeno-X Rapid Titer kit (BD Biosciences Clontech, PaloAlto, CA, USA) according to the manufacturer’s instructions.

**Virus infections to cells or animals**

Cells were infected with adenovirus overnight in antibiotics-free medium supplemented with 2% FBS. The cells were then washed and incubated in fresh medium for an additional 12-hour before experiments. For infecting mice, AAV9 containing WT-GCH1 or MT-GCH1 cDNA was injected via tail vein under pressure in 1 ml of PBS with 1 X 10^11^ IFUs of loaded virus. The concentration of DNA was 10 mg/kg.

**Western blotting**

As described previously[13], aortic tissues were homogenized on ice in cell-lysis buffer (20 mM Tris-HCl, pH 7.5, 150 mM NaCl, 1 mM Na_2_EDTA, 1 mM EGTA, 1% Triton, 2.5 mM sodium pyrophosphate, 1 mM beta-glycerophosphate, 1 mM Na_3_VO_4_, 1 µg/ml leupeptin) and 1 mM PMSF. Cell was lysated with cell-lysis buffer. The protein content was assayed by BCA protein assay reagent (Pierce, USA). 20 µg proteins were loaded to SDS-PAGE and then transferred to membrane. Membrane was incubated with a 1:1000 dilution of primary antibody, followed by a 1:2000 dilution of horseradish peroxidase- conjugated secondary antibody. Protein bands were visualized by ECL (GE Healthcare). The intensity (area X density) of the individual bands on Western blots was measured by densitometry (model GS-700, Imaging Densitometer; Bio-Rad). The background was subtracted from the calculated area. We used control as 100%. For detection of eNOS dimer/monomer distribution, the sample was not boiled and SDS-PAGE was performed in low-temperature.

**Measurements of oxidative stress *in vitro* and *in vivo***

As recommended by Michael P. Murphy *et al*[14], we measured intracellular superoxide using DHE, hydrogen peroxide by Amplex Red, mitochondrial peroxynitrite levels by MitoPY1, and *in vivo* oxidative stress by F2-isoprostanes, which is a biomarker of lipid peroxidation and non-invasive marker.

**Intracellular superoxide using DHE**

Intracellular superoxide levels were measured using the DHE fluorescence as described previously[15]. Briefly, cells were incubated with DHE (10 µM) for 30 min, homogenized, and subjected to methanol extraction. HPLC was performed using a C-18 column (mobile phase: gradient of acetonitrile and 0.1% trifluoroacetic acid) to separate and quantify oxyethidium (product of DHE and O_2_^-^) and ethidium (a product of DHE auto-oxidation). Superoxide level was determined by conversion of DHE into oxyethidine.

**Hydrogen peroxide assay by Amplex Red**

The assay was performed using Amplex Red H_2_O_2_/peroxidase assay kits (Invitrogen, USA) according to the manufacturer's protocol as described previously[16]. Cells were cultured overnight in 6-well plates at a density of 3 X10^5^ cells/well. After treatments, the supernatant was collected and was immediately assayed using the kit. The samples were mixed with 0.1 mM of Amplex Red and 0.2 U/mL of horseradish peroxidase solution diluted in 1X reaction buffer. After 30’, fluorescence intensities were measured using a microplate reader at excitation/emission wavelengths of 540/590 nm (Infinite M200pro, Tecan, Switzerland).

**Detection of peroxynitrite level in mitochondria by MitoPY1**

As described previously[17], mitochondrial levels of peroxynitrite were assessed by incubating endothelial cells with MitoPY1 (10µM, Tocris, #4428). After that, cells were analyzed using a Multi-photon microscope (Leica SP8 multi-photon microscope). For analysis, z-stacks (step size: 2 µm) of 70 µm x 70 µm areas were detected at excitation wavelengths of 800 nm in multi photon mode and analyzed using Imaris software (Version 7.4.0, Bitplane). Settings were identical for all experiments. The mean grey value was calculated from 120 cells for each condition and for each of the three independent experiments.

**Measurement F_2_-isoprostanes *in vivo***

The concentration of iPF_2_α-VI in urine or blood was determined by LC-MS/MS as previously described[18]. In brief, 0.1 ml of 10 ng/ml deuterated internal standard (8iPF2α-d4; Cayman chemical, Ann Arbor, MI, USA) was added to 1 ml urine or blood. The sample was then subjected to solid phase extraction (Oasis HLB, Waters, Milford, MA, USA). The eluate was taken to dryness under a stream of nitrogen at room temperature, and afterwards redissolved in 100 μl 10% acetonitrile of which, 40 μl was injected on a reverse-phase XTerra MS C18 column (Waters, Milford, MA, USA; 3.5 μm, 2.1X100 mm). Urinary F2-isoprostanes were quantified using a Quattro Micro (Waters) mass spectrometer. To calculate the iPF_2_α-VI concentration, the analyte to internal standard peak area ratio was compared with a standard curve from 2 to 16 ng/ml iPF_2_α-VI (Cayman chemical, Ann Arbor, MI, USA). The intra-run coefficient of variation (CV) was 4.8% and the inter-run CV was 10.1%.

**Measurement of tetrahydrobiopterin (BH4)**

The levels of BH4 were determined as previously described with some modification[19]. Briefly, homogenates of aorta or cell lysates were suspended in distilled water containing 5 mM dithioerythrol, centrifuged at 12000g at 4°C for 10 min, and then subjected to oxidation in acid or base. To 100 μl aliquot of supernatant, 20 μl of 0.5 M HCl and 0.05 M iodine were added for acidic oxidation, and 20 μl of 0.5 M NaOH plus 0.05 M iodine were added for basic oxidation. After incubation for 1h in the dark at room temperature, 20 μl HCl was added to the basic oxidation only. All mixtures received 20 μl 0.1 M ascorbic acid for the reduction of excess iodine. Samples were then centrifuged for 10 min at 12000g at 4°C. Biopterin concentrations were determined by HPLC with a PR-C18 column. Elution was at a rate of 1.0 ml/min of 50 mM potassium phosphate buffer, pH 3.0. Fluorescence was detected with an excitation at 350 nm and emission at 440 nm. BH4 concentrations were calculated as the difference in results from oxidation in acid and base.

**GCH1 activity assay**

GCH1 activity was measured in tissue extracts and cell pellets by HPLC analysis after iodine oxidation as described previously[20]. In brief, snap frozen tissue samples were homogenized, and cell pellets were freeze-thawed in lysis buffer (0.1 M Tris, 0.3 M potassium chloride, 2.5 mM EDTA, 100 μM phenylmethylsulfonyl fluoride, pH 7.8). The lysates were incubated for 1 h at 37°C with 10 mM GTP in the absence of light. The samples were then oxidized with 0.1 M potassium iodide/iodine and deproteinated with 1 M HCl for 1 h at room temperature in the absence of light. The reaction was stopped by addition of 0.1 M ascorbic acid, and 16 units/ml of alkaline phosphatase was added for 1 h at 37°C in the absence of light. Neopterin content was quantified by isocratic HPLC and fluorescence detection (JASCO) detection. Quantitation of neopterin was carried out by comparison with external standards and normalized for sample protein content. The protein concentration of each sample was measured using the BCA protein assay kit (Pierce).

**Measurement of Cystathionine β-Synthase (CBS) bioactivity**

The CBS activity in cells were determined by commercial enzyme linked chemiluminescent kit according to the instructions provided by the manufacturer as described previously[21]. In brief, after adding separate solutions to the samples as instructed, the samples were read at 340 nm for every two minutes for five times.

**Immunohistochemistry (IHC)**

The tissue was fixed in 4% paraformaldehyde overnight, and then processed, embedded in paraffin, and sectioned at 4 µm. The de-paraffinized, rehydrated section (5 µm) were microwaved in citrate buffer for antigen retrieval. Sections were incubated in endogenous peroxidase (DAKO) and protein block buffer, and then with primary antibodies indicated overnight at 4°C. Slides were rinsed with washing buffer and incubated with labelled polymer-horseradish peroxidase-antimouse/antirabbit antibodies followed by DAB^+^ chromogen detection (DAKO). After final washes, sections were counterstained with hematoxylin. All positive staining was confirmed by ensuring that no staining occurred under the same conditions with the use of non-immune rabbit or mouse control IgG.

**Human subjects**

We enrolled 50 HHcy patients at the Second Affiliated Hospital (Henan Mental Hospital) of Xinxiang Medical University in 2019-2020 in this study. Inclusion criteria were as follows: (1) age ≥60 years, (2) history of diabetes ≥10 years, (3) absence of central nervous system disease, (4) without severe hearing or vision disorders, and (5) agreement for evaluation of cognitive function. The cognitive function was assessed using the Mini-Mental State Examination (MMSE)[22]. HHcy was defined as plasma level of homocysteine above 15 μM. Leucocytes were isolated from peripheral blood as described previously[23] and subjected to perform western blot analysis of GCH1 S-nitrosylation and to measure GCH1 activity. 50 human subjects without HHcy were recruited as control. The Ethics Committee of the Second Affiliated Hospital of Xinxiang Medical University approved this clinical study, and the approval included a waiver of individual consent.

**Measurements of plasma homocysteine and HTL**

The determination of HTL has been described previously[24]. Plasma levels of homocysteine were measured by highly selective analytical methods like HPLC combined with fluorescence detection as described previously[25].

**Statistical analysis**

All quantitative results were expressed as mean ± SEM. The normal distribution of data was tested by the Kolmogorov-Smirnov test before statistical comparisons, and the normality/equal variance was tested to determine whether ANOVA was appropriate. A one-way ANOVA followed by Tukey’s HSD test was used to determine *P* value between two groups in Figure 1C, 1D, 2A, 2B, 2C, 2D, 3A, 3B, 3C, 3D, 3F, 3H, 3I, 4B, 4C, 4D, 4E, 5B, 5D, 5E, 5F, 5G and 5I because N is identical in each group. A one-way ANOVA followed by **Scheffe** test was used to determine *P* value between two groups in Figure 6D and 7D, Table 1 and 2, Online Figure S2C, S2D, S3A, S3B, S3C, S3D, S4A, S4B, S4C, S5C, S5D, and Table 1-4 because N is not identical in each group. A one-way ANOVA followed by Dunnett test was used to determine *P* value between two groups in Figure 1E and 1F because other groups are only compared with control group. A repeated-measures ANOVA following by **Bonferroni correction** was used to determine *P* value between two groups in Figure 1A and 1B. An unpaired Student's *t* test was used to determine *P* value between two groups in Online Figure S6B, S6C, S6D, S6E, S6F, and S6G. Statistical analysis was conducted using IBM SPSS statistics 20.0 (IBM Corp., Armonk, NY, USA), and *P* < 0.05 was considered significant.

**References**

[1] P. Li, M.L. Zhu, G.P. Pan, J.X. Lu, F.R. Zhao, X. Jian, L.Y. Liu, G.R. Wan, Y. Chen, S. Ping, S.X. Wang, C.P. Hu Vitamin B6 prevents isocarbophos-induced vascular dementia in rats through N-methyl-D-aspartate receptor signaling. Clin Exp Hypertens 40 (2018) 192-201.

[2] T. Washio, H. Sasaki, S. Ogoh Transcranial Doppler-determined change in posterior cerebral artery blood flow velocity does not reflect vertebral artery blood flow during exercise. American journal of physiology Heart and circulatory physiology 312 (2017) H827-H831.

[3] T.J. Li, Y. Qiu, P.Y. Yang, Y.C. Rui, W.S. Chen Timosaponin B-II improves memory and learning dysfunction induced by cerebral ischemia in rats. Neuroscience letters 421 (2007) 147-151.

[4] Y. Shen, L. Hua, C.K. Yeh, L. Shen, M. Ying, Z. Zhang, G. Liu, S. Li, S. Chen, X. Chen, X. Yang Ultrasound with microbubbles improves memory, ameliorates pathology and modulates hippocampal proteomic changes in a triple transgenic mouse model of Alzheimer's disease. Theranostics 10 (2020) 11794-11819.

[5] H. Cao, C. Zuo, Z. Gu, Y. Huang, Y. Yang, L. Zhu, Y. Jiang, F. Wang High frequency repetitive transcranial magnetic stimulation alleviates cognitive deficits in 3xTg-AD mice by modulating the PI3K/Akt/GLT-1 axis. Redox Biol 54 (2022) 102354.

[6] D. Kumaran, M. Udayabanu, M. Kumar, R. Aneja, A. Katyal Involvement of angiotensin converting enzyme in cerebral hypoperfusion induced anterograde memory impairment and cholinergic dysfunction in rats. Neuroscience 155 (2008) 626-639.

[7] P. Li, Y.L. Yin, M.L. Zhu, G.P. Pan, F.R. Zhao, J.X. Lu, Z. Liu, S.X. Wang, C.P. Hu Chronic administration of isocarbophos induces vascular cognitive impairment in rats. Journal of cellular and molecular medicine 20 (2016) 731-739.

[8] Y. Yin, D. Gao, Y. Wang, Z.H. Wang, X. Wang, J. Ye, D. Wu, L. Fang, G. Pi, Y. Yang, X.C. Wang, C. Lu, K. Ye, J.Z. Wang Tau accumulation induces synaptic impairment and memory deficit by calcineurin-mediated inactivation of nuclear CaMKIV/CREB signaling. Proc Natl Acad Sci U S A 113 (2016) E3773-3781.

[9] A.L. Dinel, C. Lucas, D. Guillemet, S. Laye, V. Pallet, C. Joffre Chronic Supplementation with a Mix of Salvia officinalis and Salvia lavandulaefolia Improves Morris Water Maze Learning in Normal Adult C57Bl/6J Mice. Nutrients 12 (2020).

[10] W.W. Bai, Z.Y. Tang, T.C. Shan, X.J. Jing, P. Li, W.D. Qin, P. Song, B. Wang, J. Xu, Z. Liu, H.Y. Yu, Z.M. Ma, S.X. Wang, C. Liu, T. Guo Up-regulation of paired-related homeobox 2 promotes cardiac fibrosis in mice following myocardial infarction by targeting of Wnt5a. J Cell Mol Med 24 (2020) 2319-2329.

[11] S.N. Zhou, J.X. Lu, X.Q. Wang, M.R. Shan, Z. Miao, G.P. Pan, X. Jian, P. Li, S. Ping, X.Y. Pang, Y.P. Bai, C. Liu, S.X. Wang S-Nitrosylation of Prostacyclin Synthase Instigates Nitrate Cross-Tolerance In Vivo. Clin Pharmacol Ther 105 (2019) 201-209.

[12] A. Romero, A. San Hipolito-Luengo, L.A. Villalobos, S. Vallejo, I. Valencia, P. Michalska, N. Pajuelo-Lozano, I. Sanchez-Perez, R. Leon, J.L. Bartha, M.J. Sanz, J.D. Erusalimsky, C.F. Sanchez-Ferrer, T. Romacho, C. Peiro The angiotensin-(1-7)/Mas receptor axis protects from endothelial cell senescence via klotho and Nrf2 activation. Aging Cell 18 (2019) e12913.

[13] S. Wang, C. Zhang, M. Zhang, B. Liang, H. Zhu, J. Lee, B. Viollet, L. Xia, Y. Zhang, M.H. Zou Activation of AMP-activated protein kinase alpha2 by nicotine instigates formation of abdominal aortic aneurysms in mice in vivo. Nature medicine 18 (2012) 902-910.

[14] M.P. Murphy, H. Bayir, V. Belousov, C.J. Chang, K.J.A. Davies, M.J. Davies, T.P. Dick, T. Finkel, H.J. Forman, Y. Janssen-Heininger, D. Gems, V.E. Kagan, B. Kalyanaraman, N.G. Larsson, G.L. Milne, T. Nystrom, H.E. Poulsen, R. Radi, H. Van Remmen, P.T. Schumacker, P.J. Thornalley, S. Toyokuni, C.C. Winterbourn, H. Yin, B. Halliwell Guidelines for measuring reactive oxygen species and oxidative damage in cells and in vivo. Nat Metab 4 (2022) 651-662.

[15] S. Wang, J. Xu, P. Song, Y. Wu, J. Zhang, H. Chul Choi, M.H. Zou Acute inhibition of guanosine triphosphate cyclohydrolase 1 uncouples endothelial nitric oxide synthase and elevates blood pressure. Hypertension 52 (2008) 484-490.

[16] B.J. Day, E. Min, J. Huang, C. Stanley The Use of Thiocyanate Formulations to Create Manganese Porphyrin Antioxidants That Supplement Innate Immunity. Antioxidants (Basel) 11 (2022).

[17] P. Kameritsch, M. Singer, C. Nuernbergk, N. Rios, A.M. Reyes, K. Schmidt, J. Kirsch, H. Schneider, S. Muller, K. Pogoda, R. Cui, T. Kirchner, C. de Wit, B. Lange-Sperandio, U. Pohl, M. Conrad, R. Radi, H. Beck The mitochondrial thioredoxin reductase system (TrxR2) in vascular endothelium controls peroxynitrite levels and tissue integrity. Proc Natl Acad Sci U S A 118 (2021).

[18] E.L. Larsen, A. Weimann, H.E. Poulsen Interventions targeted at oxidatively generated modifications of nucleic acids focused on urine and plasma markers. Free Radic Biol Med 145 (2019) 256-283.

[19] T. Fukushima, J.C. Nixon Analysis of reduced forms of biopterin in biological tissues and fluids. Anal Biochem 102 (1980) 176-188.

[20] A.L. Tatham, M.J. Crabtree, N. Warrick, S. Cai, N.J. Alp, K.M. Channon GTP cyclohydrolase I expression, protein, and activity determine intracellular tetrahydrobiopterin levels, independent of GTP cyclohydrolase feedback regulatory protein expression. J Biol Chem 284 (2009) 13660-13668.

[21] H. Wang, Q. Sun, Y. Zhou, H. Zhang, C. Luo, J. Xu, Y. Dong, Y. Wu, H. Liu, W. Wang Nitration-mediated deficiency of cystathionine beta-synthase activity accelerates the progression of hyperhomocysteinemia. Free Radic Biol Med 113 (2017) 519-529.

[22] K. Yoshiyama, H. Arita, J. Suzuki The Effect of Aroma Hand Massage Therapy for People with Dementia. J Altern Complement Med 21 (2015) 759-765.

[23] L. Nimrichter, M.M. Burdick, K. Aoki, W. Laroy, M.A. Fierro, S.A. Hudson, C.E. Von Seggern, R.J. Cotter, B.S. Bochner, M. Tiemeyer, K. Konstantopoulos, R.L. Schnaar E-selectin receptors on human leukocytes. Blood 112 (2008) 3744-3752.

[24] H. Jakubowski The determination of homocysteine-thiolactone in biological samples. Analytical biochemistry 308 (2002) 112-119.

[25] D.E. Smith, Y.M. Smulders, H.J. Blom, J. Popp, F. Jessen, A. Semmler, M. Farkas, M. Linnebank Determinants of the essential one-carbon metabolism metabolites, homocysteine, S-adenosylmethionine, S-adenosylhomocysteine and folate, in cerebrospinal fluid. Clinical chemistry and laboratory medicine : CCLM / FESCC 50 (2012) 1641-1647.

**
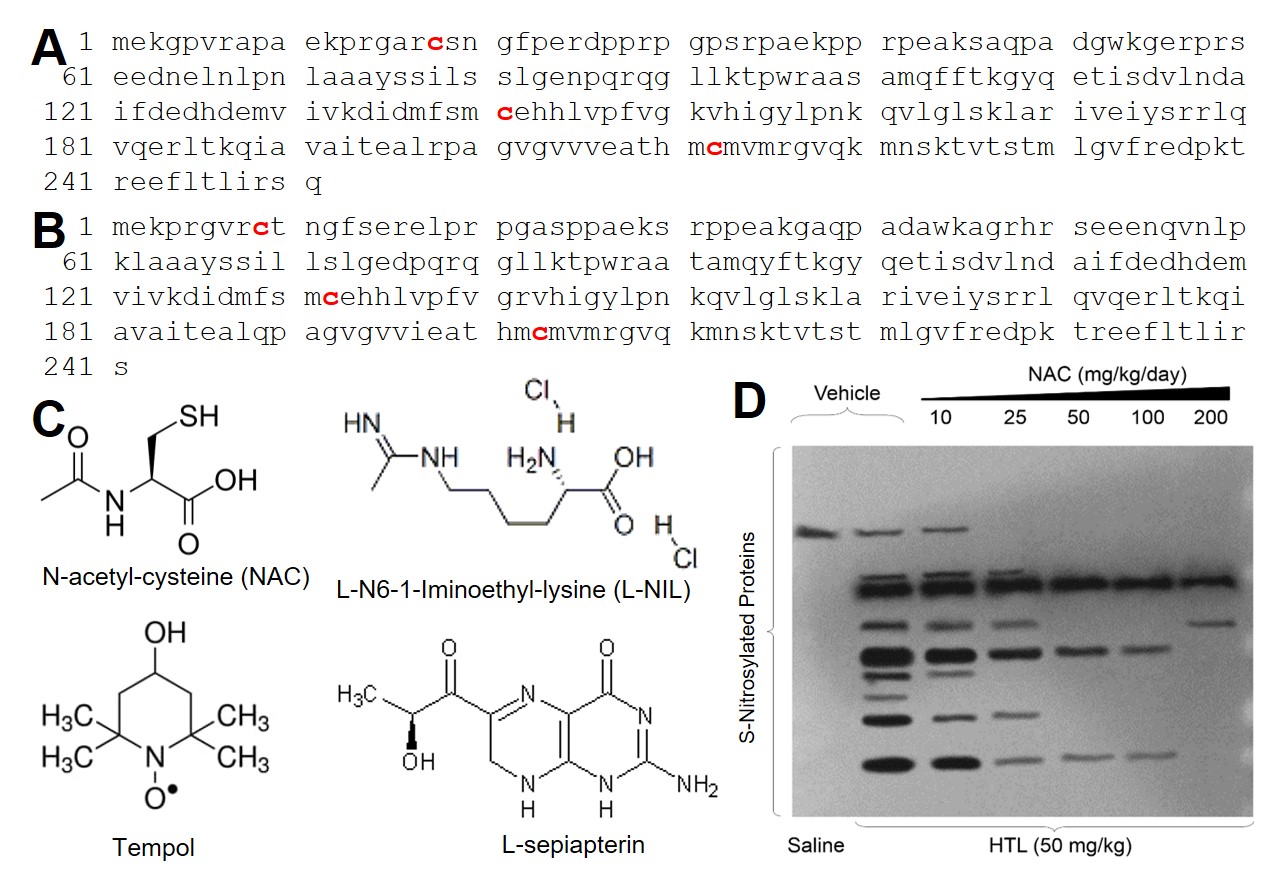
**

**Online Figure S1. The amino acid sequences of GCH1 proteins from human and mouse**. (**A**) Human GCH1 protein contains 3 cysteine residues, which locate in the 18^th^, 141^th^, and 212^th^ of amino acids. (**B**) Murine GCH1 protein contains 3 cysteine residues, which locate in the 9^th^, 132^th^, and 203^th^ of amino acids. (**C**) Chemical structures of N-acetyl-cysteine (NAC), L-N6-1-Iminoethyl-lysine (L-NIL), tempol, and L-sepiapterin. (**D**) Inhibition of NAC on protein S-tnitrosylation *in vivo*. Mice received HTL (100 mg/kg/day, 1 ml/kg per two days) administration for 16 consecutive weeks intragastrically with NAC (10, 25, 50, 100, 200 mg/kg/day) in the drinking water. Homogenates of MCA isolated from mice were subjected to measure protein S-nitrosylation using the “Biotin-switch” method.


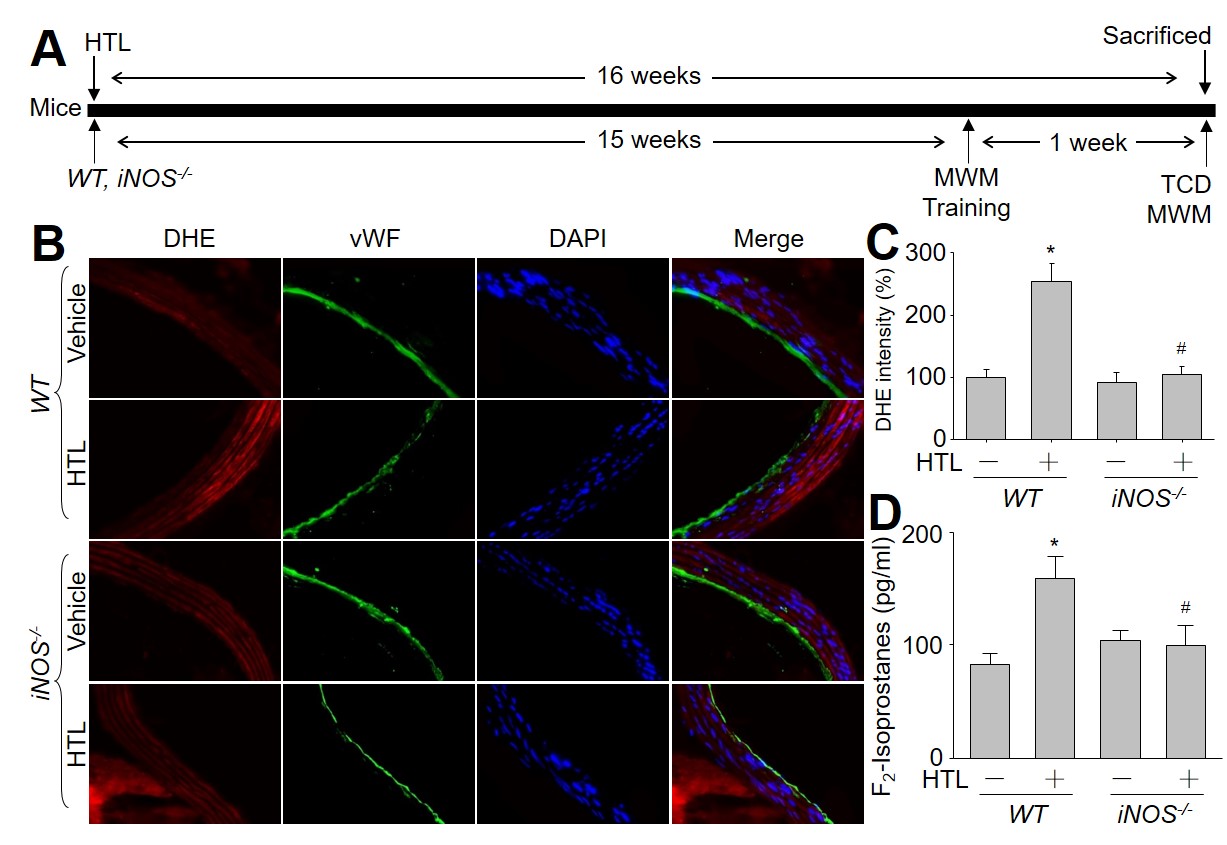


**Online Figure S2. Homocysteine thiolactone (HTL) induces oxidative stress in wildtype (*WT*) mice, but not in gene knockout of iNOS (*iNOS^-/-^*) mice**. (**A**) *WT* and *iNOS^-/-^* mice were treated with HTL (100 mg/kg/day) for 16 consecutive weeks. Before sacrifice, MCA flow velocity and PWV were measured by TCD. The cognitive functions were assessed by step-down avoidance test, passive avoidance step-through task test, and MWM test. The representative swimming traces of MWM test were shown in Figure 6A. The TCD pictures were shown in Figure 6B. Quantitative analyses about MWM test and MCA flow velocity were summarized in Table 2. (**B**) Left common carotid arteries isolated from mice were used for determining ROS productions by DHE fluorescence. Red, DHE; Green, vWF; Blue, DAPI. (**C**) Quantitative analyses of pictures in **B** were represented. (**D**) Blood levels of F_2_-isoprostanes. N = 10-15 per group. ^*^*P* < 0.05 *vs. WT*. ^#^*P* < 0.05 *vs. WT* plus HTL. A one-way ANOVA followed by **Scheffe** test was used to determine *P* value between two groups in **C** and **D**.

**
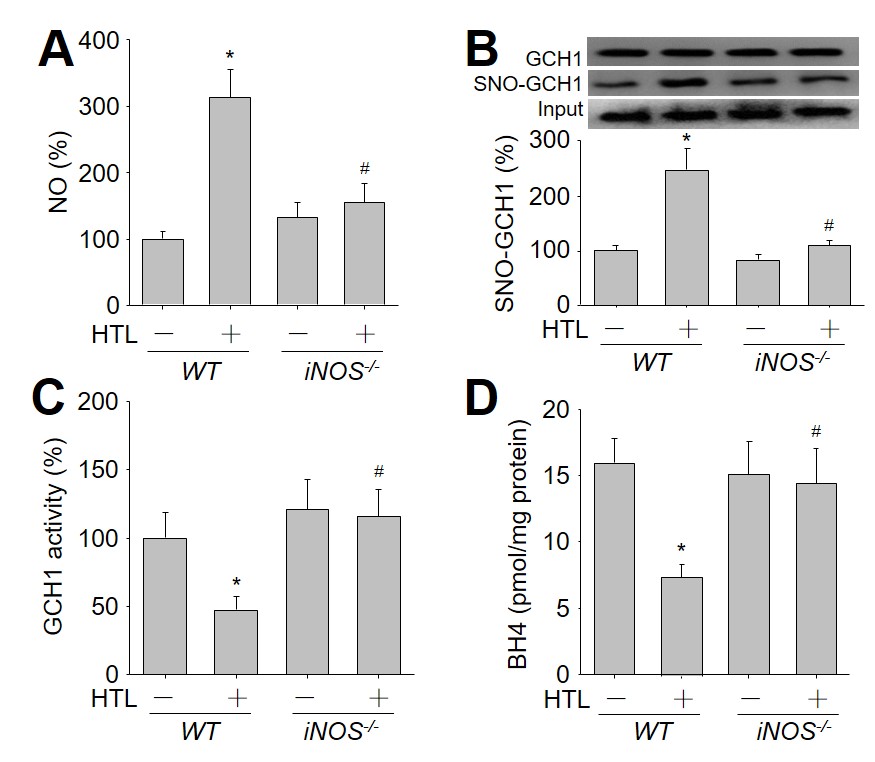
**

**Online Figure S3. Homocysteine thiolactone (HTL) induces GCH1 S-nitrosylation in wildtype (*WT*) mice, but not in gene knockout of iNOS (*iNOS^-/-^*) mice**. The experimental protocols were described and graphical in Online Figure S2A. Homogenates of right common carotid arteries were subjected to measure NO levels in **A**, GCH1 S-nitrosylation in **B**, GCH1 activity in **C**, and BH4 contents in **D**. N = 10-15 per group. ^*^*P* < 0.05 *vs. WT*. ^#^*P* < 0.05 *vs.* WT plus HTL. A one-way ANOVA followed by **Scheffe** test was used to determine *P* value between two groups in this figure.

**
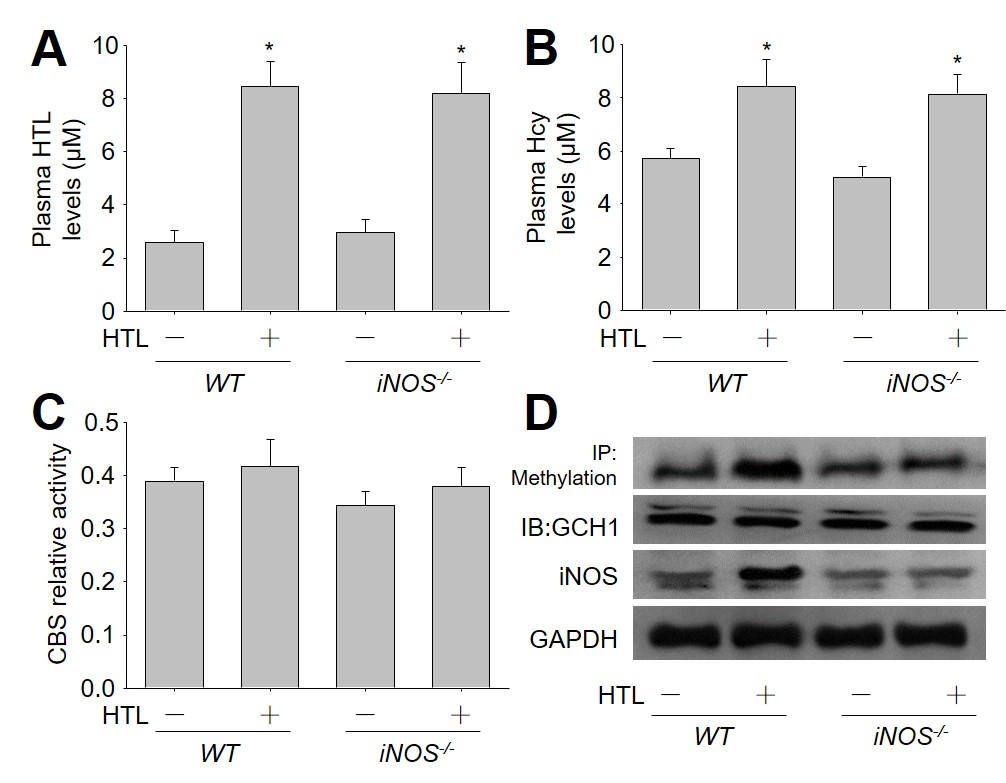
**

**Online Figure S4. Administration of homocysteine thiolactone (HTL) mimics the model of hyperhomocysteinemia in wildtype (*WT*) and gene knockout of iNOS (*iNOS^-/-^*) mice**. The experimental protocols were described and graphical in Online Figure S2A. Blood or while blood cell was collected to measure the levels of HTL in **A**, homocysteine (Hcy) in **B**, and cystathionine β-synthase (CBS) activity in **C**, and protein mono-methylation in **D**. N = 10-15 per group. ^*^*P* < 0.05 *vs.* HTL plus WT. A one-way ANOVA followed by **Scheffe** test was used to determine *P* value between two groups in **A**, **B** and **C**.


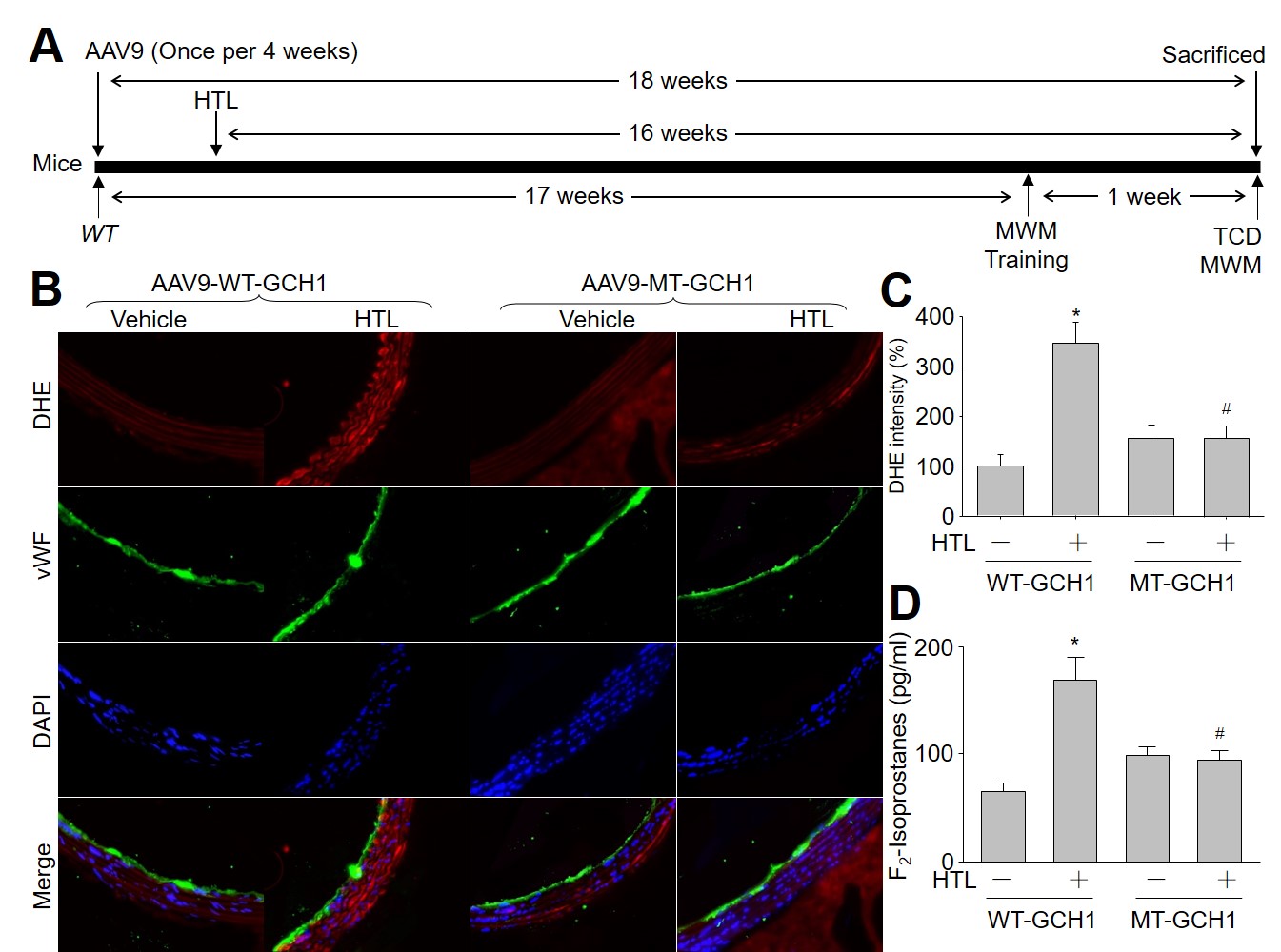


**Online Figure S5. Exogenous expression of S-nitrosylation-resistant GCH1 (MT-GCH1) prevents oxidative stress in mice fed with homocysteine thiolactone (HTL)**. (**A**) Wildtype (*WT*) mice were infected with AAV9 expressing WT-GCH1 or MT-GCH1 (C141A) via tail vein injection (once per 4 weeks) followed by 16-week HTL administration (100 mg/kg/day). Before sacrifice, MCA flow velocity and PWV were measured by TCD. The cognitive functions were assessed by step-down avoidance test, passive avoidance step-through task test, and MWM test. The representative swimming traces of MWM test were shown in Figure 7A. The TCD pictures were shown in Figure 7B. Quantitative analyses about MWM test and MCA flow velocity were summarized in Table 4. (**B**) Left common carotid arteries isolated from mice were used for determining ROS productions by DHE fluorescence. Red, DHE; Green, VWF; Blue, DAPI. (**C**) Quantitative analyses of pictures in **B** were represented. (**D**) Blood levels of F_2_-isoprostanes. N = 10-15 per group. **P*<0.05 *vs.* WT-GCH1 alone. ^#^*P*<0.05 *vs.* MT-GCH1. A one-way ANOVA followed by **Scheffe** test was used to determine *P* value between two groups in **C** and **D**.


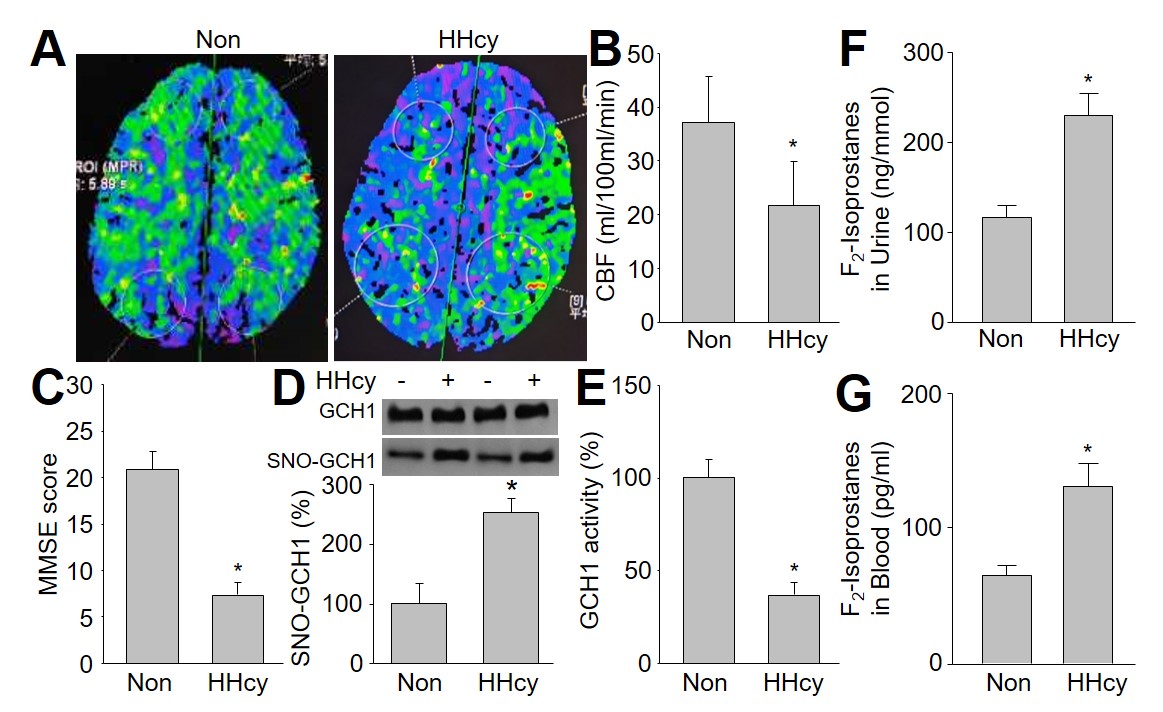


**Online Figure S6. Increased GCH1 S-nitrosylation and impaired cognitive functions in patients with HHcy**. 50 healthy human subjects and 50 HHcy patients were recruited. (**A** and **B**) Cerebral blood flow was determined by computed tomography perfusion imaging in **A** and calculated as ml per 100 ml tissue in a minute (ml/100ml/min) in **B**. (**C**) MMSE score was calculated to assess cognitive function. (**D** and **E**) Total cell lysates of leucocytes from peripheral blood were subjected to determine GCH1 S-nitrosylation by biotin-switched method in **D** and GCH1 activity in **E**. (**F**) Urine levels of F_2_-isoprostanes. (**G**) Blood levels of F_2_-isoprostanes. **P*<0.05 *vs.* non. An unpaired Student's *t* test was used to determine *P* value between two groups in **B** to **G**.
